# Cyclometalated iridium(iii) complexes as lysosome-targeted photodynamic anticancer and real-time tracking agents†
†Electronic supplementary information (ESI) available: Synthesis and characterization data, biological studies, supplementary figures, tables and movies. CCDC 883318 and 1061866. For ESI and crystallographic data in CIF or other electronic format see DOI: 10.1039/c5sc01955a


**DOI:** 10.1039/c5sc01955a

**Published:** 2015-07-22

**Authors:** Liang He, Yi Li, Cai-Ping Tan, Rui-Rong Ye, Mu-He Chen, Jian-Jun Cao, Liang-Nian Ji, Zong-Wan Mao

**Affiliations:** a MOE Key Laboratory of Bioinorganic and Synthetic Chemistry , School of Chemistry and Chemical Engineering , Sun Yat-sen University , Guangzhou 510275 , P. R. China . Email: tancaip@mail.sysu.edu.cn ; Email: cesmzw@mail.sysu.edu.cn

## Abstract

Stimuli-activatable photosensitizers (PSs) are highly desirable for photodynamic therapy (PDT) to selectively demolish tumor cells. On the other hand, lysosomes are emerging as attractive anticancer targets. Herein, four cyclometalated iridium(iii)–β-carboline complexes with pH-responsive singlet oxygen (^1^O_2_) production and lysosome-specific imaging properties have been designed and synthesized. Upon visible light (425 nm) irradiation, they show highly selective phototoxicities against cancer cells. Notably, complex **2** ([Ir(N^C)_2_(N^N)](PF_6_) in which N^C = 2-phenylpyridine and N^N = 1-(2-benzimidazolyl)-β-carboline) displays a remarkably high phototoxicity index (PI = IC_50_ in the dark/IC_50_ in light) of >833 against human lung carcinoma A549 cells. Further studies show that **2**-mediated PDT induces caspase-dependent apoptosis through lysosomal damage. The pH-responsive phosphorescence of complex **2** can be utilized to monitor the lysosomal integrity upon PDT, which provides a reliable and convenient method for *in situ* monitoring of therapeutic effect and real-time assessment of treatment outcome. Our work provides a strategy for the construction of highly effective multifunctional subcellular targeted photodynamic anticancer agents through rational structural modification of phosphorescent metal complexes.

## Abstract 2

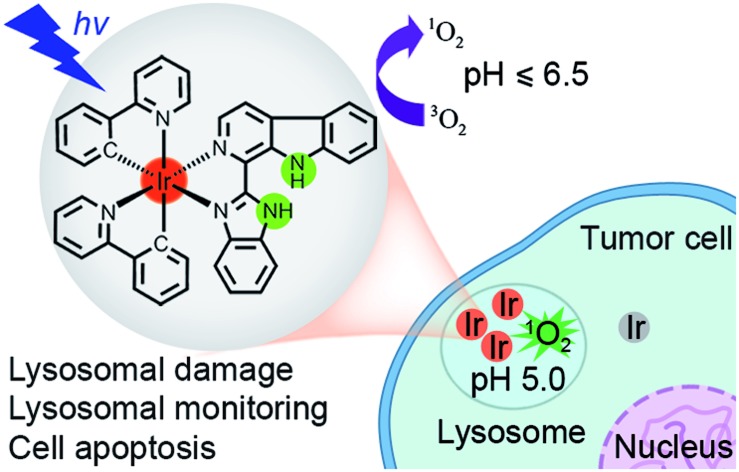
We report the rational design and photodynamic anticancer mechanism studies of iridium(iii) complexes with pH-responsive singlet oxygen (^1^O_2_) production and lysosome-specific imaging properties.

## Introduction

Iridium complexes have recently emerged as promising alternatives to platinum-based metallo-anticancer drugs.^1^ Phosphorescent cyclometalated iridium(iii) complexes provide excellent phosphorescence properties, *e.g.*, high quantum yields, large Stokes shifts, long-lived phosphorescence, outstanding color-tuning capability and good resistance to photobleaching, thus they have been widely explored as biological imaging and sensing probes.^2^ Additionally, it has been reported that iridium(iii) complexes can function as efficient photosensitizers (PSs) for producing singlet oxygen (^1^O_2_).^3^ Due to the easy modification of the ligands, the photophysical and biological properties of iridium(iii) complexes can be readily tuned.1*d* Most importantly, the integration of the anticancer potencies and the phosphorescent properties of cyclometalated iridium(iii) complexes offers opportunity for the construction of novel theranostic platforms. Although the environment-sensitive emission of phosphorescent iridium(iii) complexes is well known, they are rarely explored as stimuli-responsive photodynamic therapy (PDT) agents.

PDT is an attractive non-invasive modality for cancer treatment.^4^ Upon irradiation, the excited PS transfers its energy to the surrounding molecular oxygen (^3^O_2_) to generate ^1^O_2_ and other cytotoxic reactive oxygen species (ROS), which can damage cancer cells irreversibly.^5^ Stimuli-activatable PSs are highly desirable for PDT to improve the selectivity against cancer cells and reduce side effects.^6^ As the tumor microenvironment is more acidic (pH 6.5–6.8) than blood and normal tissues (pH *ca.* 7.4),^7^ the acidic pH-activatable PSs have attracted increasing interest.^8^

Lysosomes (pH 4.5–5.5) contain a variety of hydrolytic enzymes that are capable of degrading almost all kinds of biomolecules.^9^ Disruption of the lysosomal integrity can initiate cell death through a process known as lysosomal membrane permeabilization (LMP).^10^ Widespread LMP results in the release of cathepsins and other hydrolases from the lysosomal lumen to the cytosol. These enzymes thus initiate apoptosis by cleaving a variety of substrates including caspases and many members of the Bcl-2 protein family.^11^ As lysosomes are involved in various aspects of cell death, they are emerging as attractive pharmacological targets for selective killing of cancer cells.^12^ The pH-sensitive PSs can target tumor tissues and further be activated by the significantly increased acidity in the lysosomes of cancer cells.8*d* The real-time monitoring of lysosomal integrity and physiological status is fundamental for the understanding of lysosomal functions during the therapeutic process.

Recently, we have developed a series of iridium(iii) complexes with β-carboline (a kind of biologically active indole alkaloids) ligands as multifunctional anticancer agents.^13^ Herein, we aim to develop iridium(iii) complexes with β-carboline ligands as pH-responsive tumor/lysosome-targeted PDT agents. Imidazole and benzimidazole groups, which are reported to be important pharmacophores and pH-responsive groups, are introduced to the β-carboline ligands to enhance their selectivity toward cancer cells and sensitivity to acidic environments.8b,^14^ The mechanisms of action for PDT, the selectivity for cancer cells as well as the potential of real-time monitoring lysosomal damage during PDT of these complexes have been explored.

## Results and discussion

### Synthesis and characterization

**Fig. 1:**
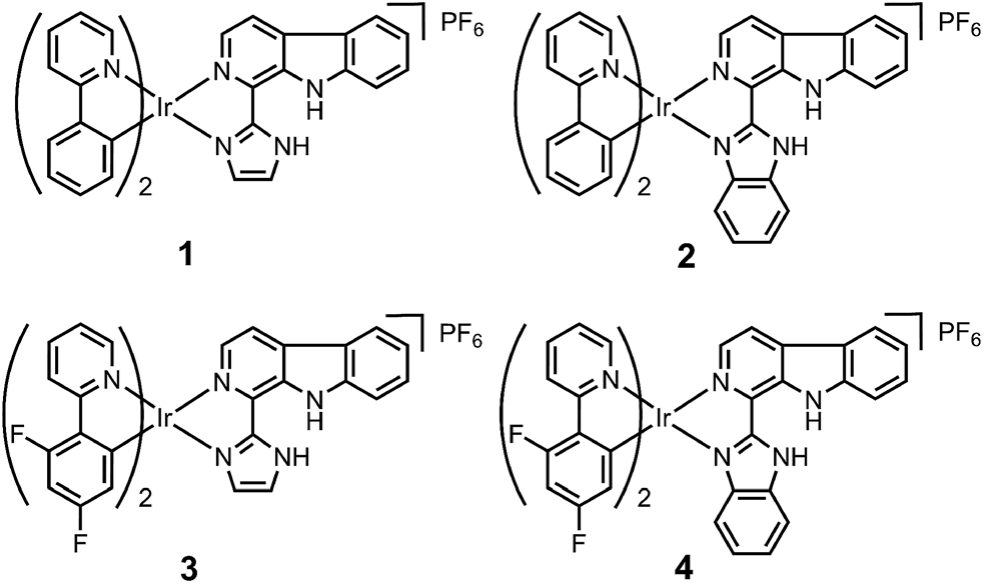
Chemical structures of iridium(iii) complexes **1–4**.

**Fig. 2:**
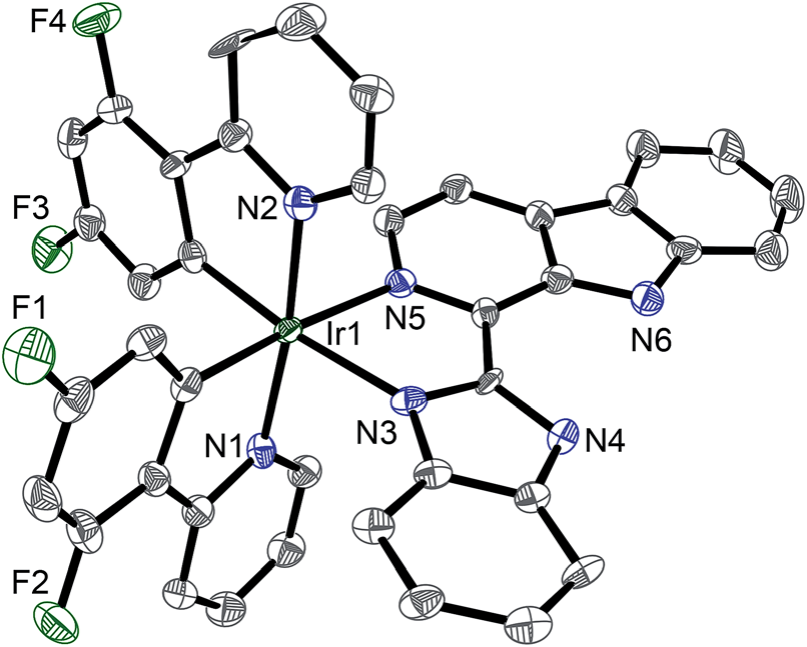
X-Ray crystal structure of **4**·2CH_3_CH_2_OH with thermal ellipsoids set at the 50% probability level. The H atoms, counterion and solvent have been omitted for clarity.

Ligand **L2** (1-(2-benzimidazolyl)-β-carboline, Fig. S1†) was synthesized by condensation of tryptamine and benzimidazole-2-carbaldehyde in anisole, similar to that described for **L1** (1-(2-imidazolyl)-β-carboline, Fig. S1†).^15^ Iridium(iii) complexes **1–4** with the general formula [Ir(N^C)_2_(N^N)](PF_6_) (Fig. 1; N^C = ppy (2-phenylpyridine) or dfppy (2-(2,4-difluorophenyl)pyridine); N^N = **L1** or **L2**) were synthesized by refluxing two equivalents of β-carboline ligands and the corresponding cyclometalated iridium(iii) dimers in CH_2_Cl_2_–CH_3_OH (2 : 1, v/v), followed by anion exchange with NH_4_PF_6_, purification by column chromatography on silica gel, and recrystallization (see ESI†).^13^ Ligand **L2** and complexes **1–4** were characterized by ^1^H NMR spectroscopy, ESI-MS and CHN elemental analysis (see ESI, Fig. S2–S6†). Ligand **L2** and complex **4** were characterized by X-ray crystallography (Fig. 2 and S7, Tables S1 and S2†).

### Photophysical properties

Complexes **1–4** exhibited intense absorption in buffer solutions, CH_3_OH and CH_3_CN in the visible light range at approximately 380–450 nm (Fig. S8 and S9†), which could be assigned to the mixed singlet and triplet metal-to-ligand charge-transfer (^1^MLCT and ^3^MLCT) and ligand-to-ligand charge-transfer (LLCT) transitions.2*b*

**Fig. 3:**
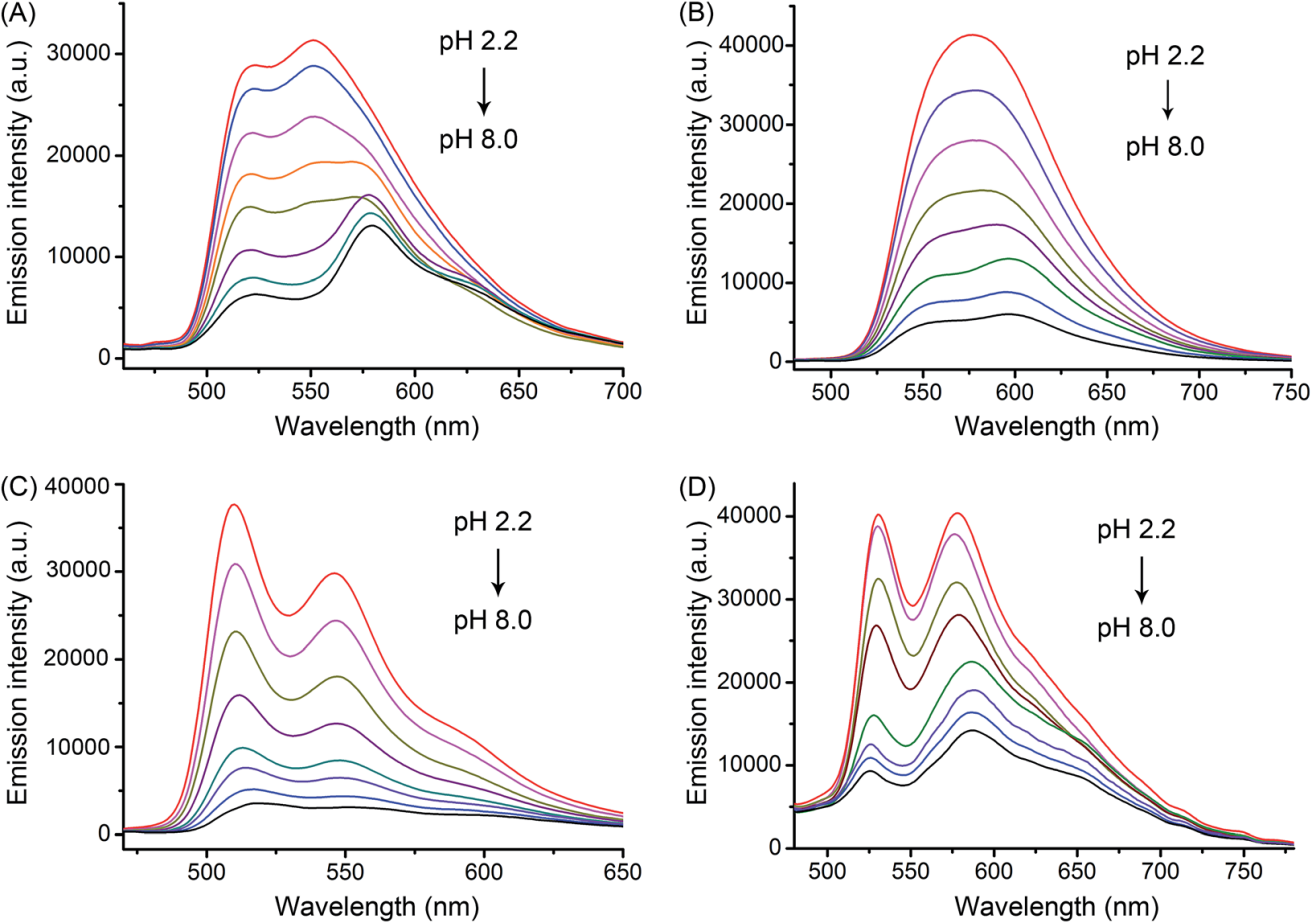
pH-sensitive emission spectra of (A) **1**, (B) **2**, (C) **3** and (D) **4** (20 μM, *λ*_ex_ = 405 nm) in disodium hydrogen phosphate/citric acid buffer solutions (pH: 2.2, 2.8, 3.4, 4.0, 5.0, 6.0, 7.0 and 8.0).

Upon excitation, complexes **1–4** exhibited pH-responsive green to orange phosphorescent emission (Fig. 3 and Table 1). The phosphorescence quantum yield (*Φ*) of **2** increased significantly from 0.019 at pH 7.4 to 0.092 at pH 3.0. Similar results were also observed for **1**, **3** and **4**. Moreover, the p*K*_a_ values determined from the phosphorescence titration curves were 3.56, 4.21, 3.62 and 4.42 for **1–4**, respectively (Fig. S10†). The pH-sensitive emission of these complexes could be contributed by the protonation/deprotonation processes of the imidazolyl/benzimidazolyl-*NH* and the indolyl-*NH* on the β-carboline ligands, which might cause pH-dependent switching from the interligand-charge-transfer (ILCT) state to highly emissive triplet ligand-to-ligand charge-transfer (^3^LLCT)/triplet metal-to-ligand charge-transfer (^3^MLCT) excited states.2h,^16^

Furthermore, complexes **1–4** displayed two-photon excited phosphorescence, which was favorable for live cell imaging and *in vivo* applications. The maximum two-photon action cross-section values (*Φδ*_max_, in which *Φ* is the phosphorescence quantum yield and *δ* is the two-photon absorption cross section) of **1–4** at acidic pH (3.0) at 810 nm were determined to be 9.9, 13.5, 8.5 and 4.1 GM, respectively.

**Table 1:** Photophysical data of complexes **1–4**

Complex	Medium pH^*a*^	*λ*_ex_/*λ*_em_^*b*^	*Φ* ^*c*^	*Φ*_Δ_ ^*d*^
**1**	3.0	405/523, 551	0.077	0.42
	5.0	405/523, 574	0.051	0.24
	6.5	405/523, 577	0.035	0.15
	7.4	405/523, 578	0.024	0.07

**2**	3.0	405/576	0.092	0.51
	5.0	405/557, 590	0.060	0.29
	6.5	405/557, 598	0.039	0.17
	7.4	405/557, 598	0.019	0.05

**3**	3.0	405/512, 547	0.063	0.31
	5.0	405/512, 548	0.034	0.14
	6.5	405/513, 548	0.022	0.09
	7.4	405/514, 549	0.014	0.04

**4**	3.0	405/527, 576	0.026	0.20
	5.0	405/526, 587	0.013	0.11
	6.5	405/525, 588	0.010	0.07
	7.4	405/525, 588	0.008	0.03

^*a*^Disodium hydrogen phosphate–citric acid buffer solutions.

^*b*^Excitation wavelengths (*λ*_ex_) and maximum wavelengths of emission spectra (*λ*_em_) in nm.

^*c*^The emission quantum yields at room temperature were determined using [Ru(bpy)_3_]Cl_2_ in aerated H_2_O (*Φ* = 0.028)^19^ as the reference.

^*d*^The *Φ*_Δ_ were determined using [Ru(bpy)_3_]Cl_2_ in aerated H_2_O (*Φ*_Δ_ = 0.18)^18^ as the reference.

**Fig. 4:**
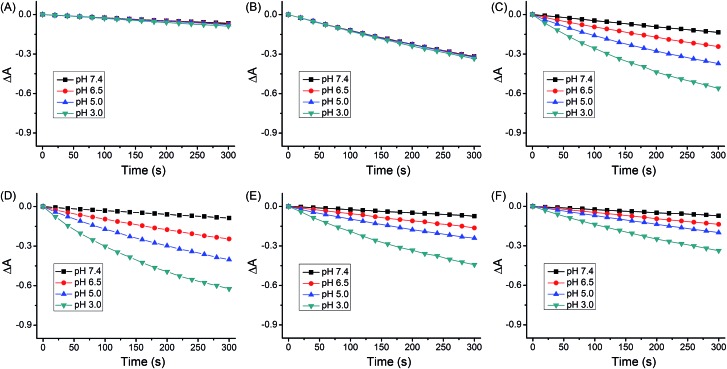
Rate of decay of ABDA sensitized by (A) control, (B) [Ru(bpy)_3_]Cl_2_, (C) **1**, (D) **2**, (E) **3** and (F) **4** in aerated disodium hydrogen phosphate/citric acid buffer solutions as shown by the decrease in the absorption maxima of ABDA (377, 378, 380 and 380 nm for pH 3.0, 5.0, 6.5 and 7.4, respectively).

The quantum yields for ^1^O_2_ production (*Φ*_Δ_) of **1–4** under light irradiation (425 nm) were evaluated in aerated buffer solutions using a steady-state method with 9,10-anthracenediyl-bis(methylene)dimalonic acid (ABDA) as the ^1^O_2_ indicator^17^ and [Ru(bpy)_3_]Cl_2_ as the standard.^18^ Unlike the *Φ*_Δ_ of [Ru(bpy)_3_]Cl_2_ (bpy = 2,2′-bipyridine), which was almost constant at different pH values, the *Φ*_Δ_ of **1–4** displayed a marked reliance on the pH of the solutions (Fig. 4 and Table 1). Notably, the *Φ*_Δ_ of **2** increased from 0.05 at pH 7.4 to 0.51 at pH 3.0. It is expected that **1–4** can photosensitize ^1^O_2_ production more efficiently in acidic environments (*e.g.*, tumor tissues and lysosomes) than at neutral pH.

### PDT activities

The cytotoxicities in the dark and PDT activities of complexes **1–4**, β-carboline ligands (**L1** and **L2**), the dimeric iridium(iii) precursors and cisplatin were assessed against human lung carcinoma A549 cells, cisplatin-resistant A549cisR cells, breast cancer MCF-7 cells and human normal liver LO2 cells (Tables 2 and S3, Fig. S11†). The complexes bearing ligand **L1** displayed moderate cytotoxicity in the dark in A549 cells, as the IC_50_ values determined for **1** and **3** were 19.5 and 29.9 μM, respectively. Whereas negligible cytotoxicity in the dark in A549 cells was observed for complexes **2** (IC_50_ ≥ 100 μM) and **4** (IC_50_ = 55.6 μM) that incorporated ligand **L2**. Similar results were also observed in A549cisR, MCF-7 and LO2 cells.

**Table 2:** (Photo)cytotoxicity (IC_50_, μM) of the tested compounds toward A549, A549cisR and LO2 cells

Compound	A549		A549cisR		LO2	
	Dark^*a*^ (light)^*b*^	PI^*c*^	Dark^*a*^ (light)^*b*^	PI^*c*^	Dark^*a*^ (light)^*b*^	PI^*c*^
**1**	19.5 ± 2.1 (0.88 ± 0.08)	22.2	18.2 ± 1.6 (0.37 ± 0.05)	49.2	24.4 ± 1.8 (9.8 ± 1.1)	2.5
**2**	>100 (0.12 ± 0.03)	>833	>100 (0.16 ± 0.03)	>625	>100 (4.4 ± 0.7)	22.7
**3**	29.9 ± 2.8 (1.2 ± 0.2)	24.9	16.1 ± 1.2 (0.47 ± 0.03)	34.3	68.7 ± 5.2 (13.4 ± 2.2)	5.1
**4**	55.6 ± 4.1 (0.81 ± 0.09)	68.6	59.4 ± 4.1 (0.14 ± 0.02)	424	87.7 ± 6.7 (10.3 ± 1.4)	8.5
**L1**	>100 (17.6 ± 1.9)	>5.7	42.6 ± 3.8 (19.7 ± 1.7)	2.2	>100 (47.4 ± 6.1)	>2.1
**L2**	>100 (>100)	—	>100 (>100)	—	>100 (>100)	—
Ir_2_(ppy)_4_Cl_2_	19.6 ± 2.6 (11.4 ± 1.4)	1.7	20.2 ± 2.3 (7.0 ± 0.5)	2.9	28.5 ± 2.4 (21.3 ± 1.6)	1.3
Ir_2_(dfppy)_4_Cl_2_	11.5 ± 1.5 (9.3 ± 1.0)	1.2	4.3 ± 0.5 (4.2 ± 0.4)	1.0	29.6 ± 3.1 (21.5 ± 2.7)	1.4
Cisplatin	23.3 ± 2.6 (22.6 ± 2.9)	1.0	85.5 ± 5.3 (86.1 ± 6.2)	1.0	31.2 ± 2.7 (30.5 ± 3.1)	1.0

^*a*^Cells were incubated with the indicated compounds for 48 h.

^*b*^Cells were incubated with the indicated compounds for 12 h and then irradiated with a 425 nm LED light array for 15 min (36 J cm^–2^).

^*c*^PI (phototoxicity index) is the ratio of the IC_50_ value in the dark to that obtained upon light irradiation.

All iridium(iii) complexes displayed relatively high phototoxicities upon visible light irradiation (425 nm, 36 J cm^–2^) against the cancer cells tested.3*f* A very high phototoxicity index (PI) of >833 was observed for complex **2** in A549 cells. The high phototoxicities of **1–4** were retained in A549cisR cells, which indicated that the PDT activity was able to bypass cisplatin resistance. Interestingly, these complexes displayed high selectivity for cancer cells tested over non-cancerous human normal liver LO2 cells (Table 2, Fig. S11 and Table S3†). Remarkably, complex **2** showed over 36-fold higher phototoxicity against A549 cells (IC_50_ = 0.12 μM and PI > 833) than against LO2 cells (IC_50_ = 4.4 μM and PI = 22.7). The PDT activities of β-carboline ligands (**L1** and **L2**), the dimeric iridium(iii) precursors and cisplatin were barely detectable.

### Lipophilicity, cellular uptake and subcellular localization

It has been reported that the cellular uptake levels of iridium complexes are influenced by many factors, *e.g.*, molecular size, lipophilicity, water-solubility and uptake mechanisms.^20^ The log *P*_o/w_ (partition coefficient in oil/water) values determined for **1–4** were 1.97, 2.07, 2.01 and 2.12, respectively (Table S4†). The cellular uptake efficiency of **1–4** was studied by inductively coupled plasma mass spectrometry (ICP-MS; Table S4†). These complexes showed similar lipophilicity, but higher uptake levels were observed for **1** and **3** than those obtained for **2** and **4**, which suggested that smaller size of the ancillary ligand might be advantageous for the cell-penetrating abilities of these iridium(iii) complexes. Nevertheless, slowly internalized molecules seem to exhibit lower cytotoxicity and tend to accumulate more in tumors than normal tissues, which may be an advantage for the development of potential photosensitizers for PDT.3e,^21^

**Fig. 5:**
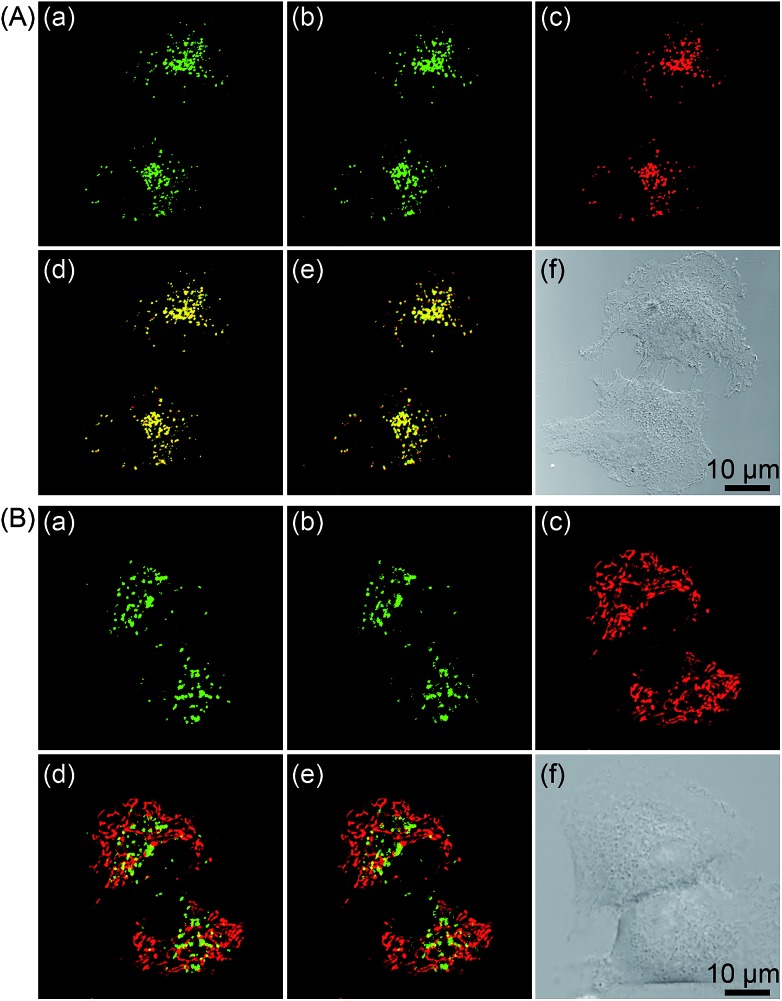
(A) One- and two-photon excited phosphorescent images of A549 cells co-labeled with **2** (20 μM, 5 h) and LTDR (150 nM, 0.5 h). (a) One-photon excited **2** (*λ*_ex_ = 405 nm, *λ*_em_ = 580 ± 30 nm). (b) Two-photon excited **2** (*λ*_ex_ = 810 nm, *λ*_em_ = 580 ± 30 nm). (c) LTDR (*λ*_ex_ = 633 nm, *λ*_em_ = 668 ± 20 nm). (d) Overlay of (a) and (c). (e) Overlay of (b) and (c). (f) Bright field. (B) One- and two-photon excited phosphorescent images of A549 cells co-labeled with **2** (20 μM, 5 h) and MTDR (150 nM, 0.5 h). (a) One-photon excited **2** (*λ*_ex_ = 405 nm, *λ*_em_ = 580 ± 30 nm). (b) Two-photon excited **2** (*λ*_ex_ = 810 nm, *λ*_em_ = 580 ± 30 nm). (c) MTDR (*λ*_ex_ = 633 nm, *λ*_em_ = 665 ± 20 nm). (d) Overlay of (a) and (c). (e) Overlay of (b) and (c). (f) Bright field.

The subcellular localization of **1–4** in A549 cells was also investigated using both one- and two-photon microscopy (Fig. 5 and S12–S14†). High Pearson's colocalization coefficients (0.82–0.89) were observed by colabeling **1–4** with the lysosome-specific fluorescent dye LysoTracker Deep Red (LTDR) in A549 cells under one- and two-photon excitation. Meanwhile, negligible colocalization was observed for **1–4** with the mitochondria-specific probe MitoTracker Deep Red (MTDR). These results indicate that **1–4** can specifically label lysosomes.

Furthermore, incubation of A549 cells with **2** at lower temperature (4 or 25 °C) or upon pretreatment with the energy inhibitor carbonyl cyanide *m*-chlorophenyl hydrazone (CCCP) resulted in reduced cellular uptake efficiency as revealed by confocal microscopy (Fig. S15†) and flow cytometry (Fig. S16†), which suggested that cellular uptake of **2** was mainly through an energy-dependent mechanism.

### pH-dependent emission in A549 cells

**Fig. 6:**
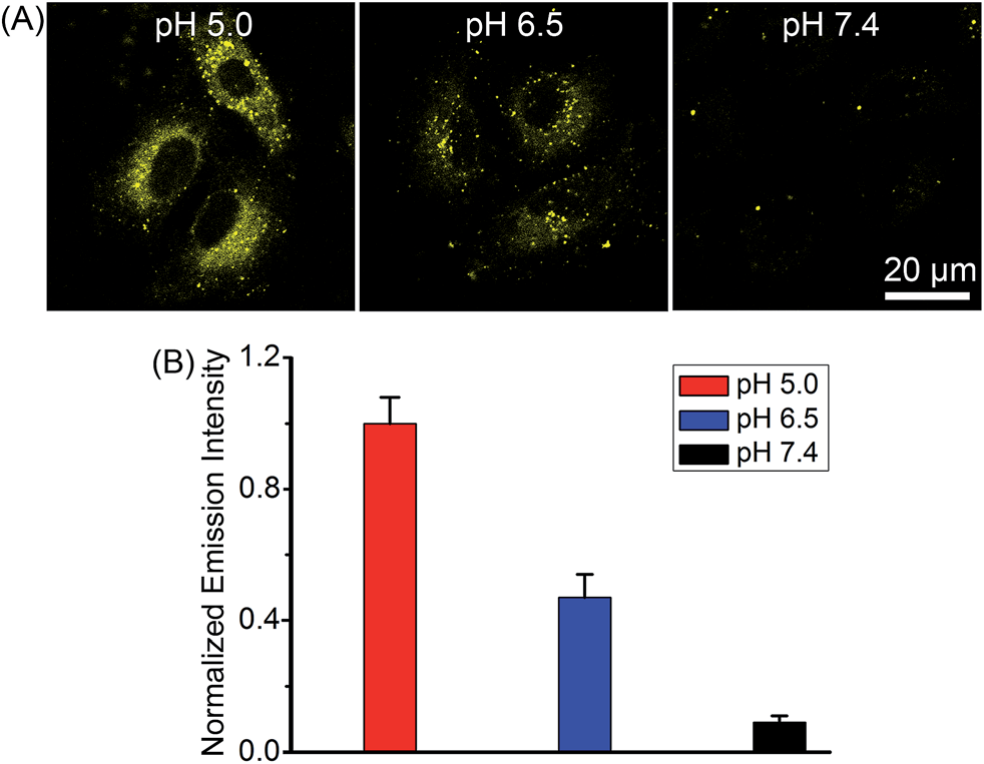
(A) pH-dependent phosphorescent images of **2**-labeled A549 cells. The cells were incubated with **2** (10 μM, *λ*_ex_ = 405 nm, *λ*_em_ = 580 ± 30 nm) for 12 h, and then treated with nigericin solutions (20 μM) at pH 5.0, 6.5 and 7.4 for 10 min. (B) Comparison of the intracellular phosphorescence intensity of **2** in the presence of nigericin at different pH values. Data are expressed as the mean ± SD (standard deviations). Number of cells: 10.

Complex **2** was chosen as the model compound to examine the pH-dependent emission in A549 cells by equilibrating intra- and extracellular pH with the ionophore nigericin.^22^ The intracellular phosphorescence intensities of **2** at pH roughly mimicking lysosomal environment (5.0) and cancer tissues (6.5) were much stronger than that obtained at pH 7.4 (Fig. 6), which demonstrated the potential of **2** for tumor/lysosome-selective imaging.

### PDT-induced apoptosis/necrosis

**Fig. 7:**
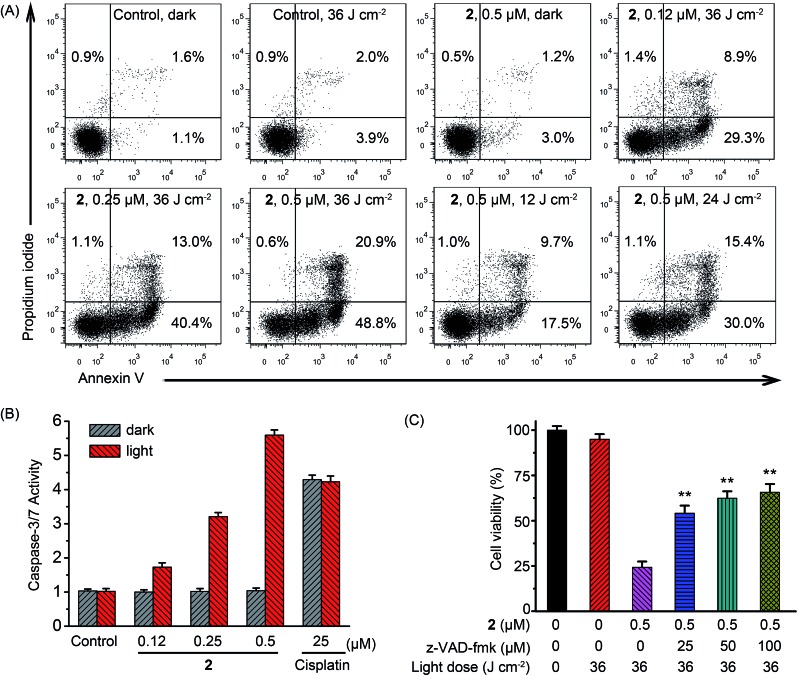
(A) Flow cytometric quantification of annexin V and propidium iodide double-labeled A549 cells treated with **2** for 24 h in the absence or presence of light at the indicated concentrations. After A549 cells were incubated with vehicle or **2** for 12 h, the cells were irradiated with a 425 nm LED light for 15 min (36 J cm^–2^). (B) Detection of caspase-3/7 activity in A549 cells after treated with **2** or cisplatin in the absence or presence of light at the indicated concentrations. Dark: cells were incubated with **2** or cisplatin for 24 h in the dark. Light: cells were incubated with **2** or cisplatin for 12 h in the dark and then irradiated with light at 425 nm (36 J cm^–2^) and further incubated for 12 h in the dark. (C) z-VAD-fmk concentration-dependent inhibition against A549 cell death induced by **2**-mediated PDT. Data are represented as means ± SD of three independent experiments. **P < 0.01, as compared with the group treated with **2** (0.5 μM) in the absence of z-VAD-fmk.

Under light irradiation, complex **2** exhibited the highest photodynamic activities in A549 cells, so it was chosen as the model compound to explore the mechanism of the phototoxicity. Lysosomes are found to be involved in the apoptotic pathways and lysosomal damage can promote apoptosis.^23^ Apoptosis induced by the **2**-mediated PDT was first verified quantitatively by flow cytometric analysis of A549 cells double-labeled with annexin V and propidium iodide.^24^ As shown in Fig. 7A, most of the cells treated with vehicle (1% DMSO (dimethyl sulfoxide)), light (36 J cm^–2^) or **2** (0.5 μM) alone remained viable with a cell mortality rate < 7%. Apoptotic cells significantly increased in PS concentration- and light dose-dependent manners upon PDT treatment. The percentage of apoptotic cells increased to 69.7% after the cells were treated with **2** (0.5 μM) in combination with light (36 J cm^–2^).

It could also be seen from confocal microscopic analysis that when A549 cells were treated with relatively high concentration of **2** (0.5 μM, *ca.* 4 times of IC_50_ value (0.12 μM)), some of the cells are in the late apoptotic/necrotic phase as they were positively stained by both annexin V and propidium iodide (Fig. S17†). It has been reported that high dose PDT (either a high PS concentration or a high light dose or both) could cause cell death by necrosis.^25^

Changes in cell morphology upon **2**-mediated PDT were further examined by 2′-(4-ethoxyphenyl)-5-(4-methyl-1-piperazinyl)-2,5′-bi-1*H*-benzimidazole trihydrochloride (Hoechst 33342) staining (Fig. S18†). Control cells displayed normal morphology with round and homogeneous nuclei. While PDT treatment caused a concentration-dependent increase in the fraction of cells showing distinct apoptotic features. After treatment of cells with **2** (0.5 μM) in combination with light (36 J cm^–2^), most of the cells displayed typical morphological changes of apoptosis, *e.g.*, plasma membrane blebbing, fragmented nuclei and apoptotic bodies.^26^ These results reveal that **2**-mediated PDT mainly kills A549 cells *via* apoptosis.

The activation of caspases has been identified as one of the key events in apoptosis.^27^ As compared with the control cells in the dark, negligible increase in caspase-3/7 activity was detected in cells treated with light irradiation or **2** alone, while the combination of **2** (0.5 μM) and light (36 J cm^–2^) caused an approximately 5.7-fold increase in caspase-3/7 activity (Fig. 7B). Light irradiation showed no effect on caspase-3/7 activation induced by cisplatin. Furthermore, z-VAD-fmk, a pan-caspase inhibitor, could efficiently attenuate cell death caused by **2**-mediated PDT (Fig. 7C). In the presence of z-VAD-fmk (100 μM), the percentage of cell viability increased from 25.3 ± 3.4% to 65.7 ± 4.5% when cells were treated with **2** (0.5 μM) upon light irradiation. These results indicate that apoptotic cell death induced by **2**-mediated PDT occurs through caspase-dependent mechanisms.

### Cellular ROS production

**Fig. 8:**
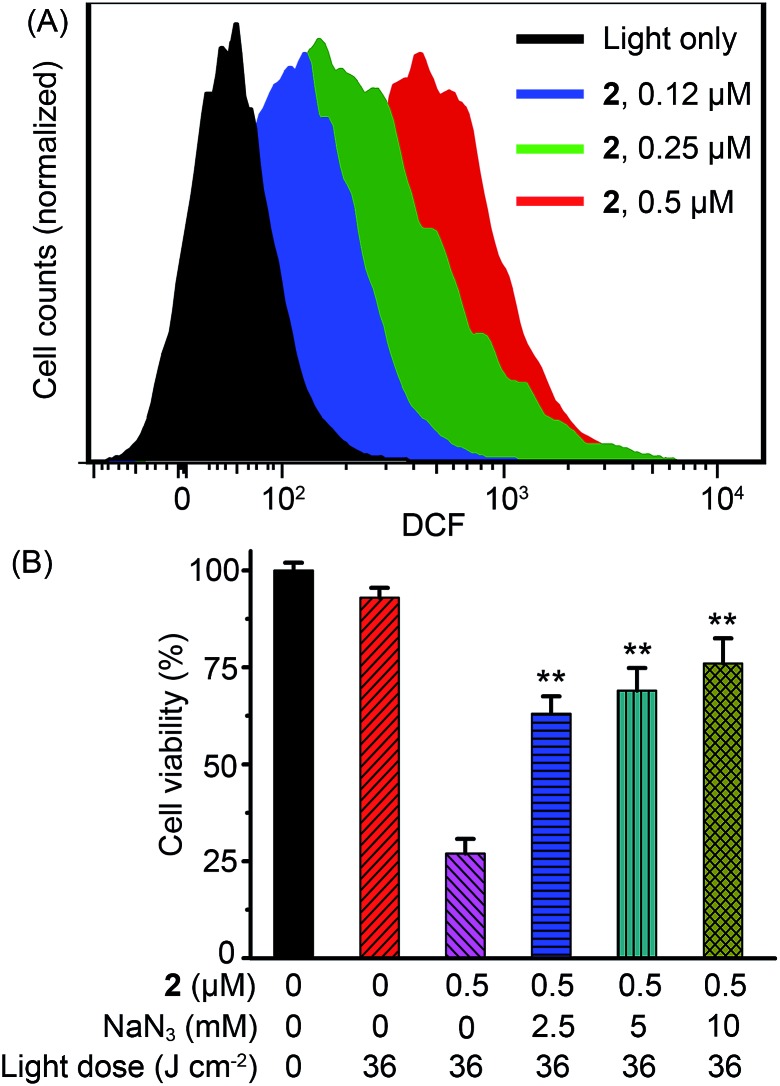
(A) Analysis of ROS generation by flow cytometry during **2**-mediated PDT with a ROS probe, DCF-DA. A549 cells were irradiated with a 425 nm LED light for 15 min (36 J cm^–2^). (B) Concentration-dependent inhibition of NaN_3_ on cell death induced by **2**-mediated PDT in A549 cells. Data are represented as means ± SD of three independent experiments. **P < 0.01, as compared with the group treated with **2** (0.5 μM) in the absence of NaN_3_.

It has been demonstrated in numerous studies that generation of ROS is the main mechanism responsible for the PDT-initiated cell death.3e,f,h,8d The intracellular production of ROS during **2**-mediated PDT was probed using the dichlorofluorescein diacetate (DCF-DA) fluorescence assay. DCF-DA is a nonfluorescent cell-permeable dye, but it undergoes oxidation by a wide range of ROS to form 2′,7′-dichlorofluorescein (DCF), which is highly fluorescent.^28^ Flow cytometric analysis showed a concentration-dependent increase in DCF fluorescence in A549 cells treated with **2** under irradiation, indicating the generation of ROS (Fig. 8A). A *ca.* 17-fold increase in the mean fluorescence intensity of DCF was observed in A549 cells treated with **2** (0.5 μM) in combination with light (36 J cm^–2^) as compared with the control cells treated with light alone. Moreover, pretreatment of cells with sodium azide (NaN_3_), an efficient physical quencher of ^1^O_2_,^29^ led to marked inhibition of apoptotic cell death, as indicated by annexin V/propidium iodide double staining (Fig. S17†) and Hoechst 33342 staining (Fig. S18†). Similarly, pretreatment of NaN_3_ significantly diminished the PDT effect of complex **2** on A549 cells (Fig. 8B). When cells were treated with **2** (0.5 μM) upon light irradiation, the percentages of viable cells in the absence and presence of NaN_3_ (10 mM) were 26.8 ± 3.8% and 76.1 ± 6.5%, respectively. These results suggest that ^1^O_2_ plays a predominant role in apoptotic cell death induced by **2**-mediated PDT.

### LMP and cathepsin B release

**Fig. 9:**
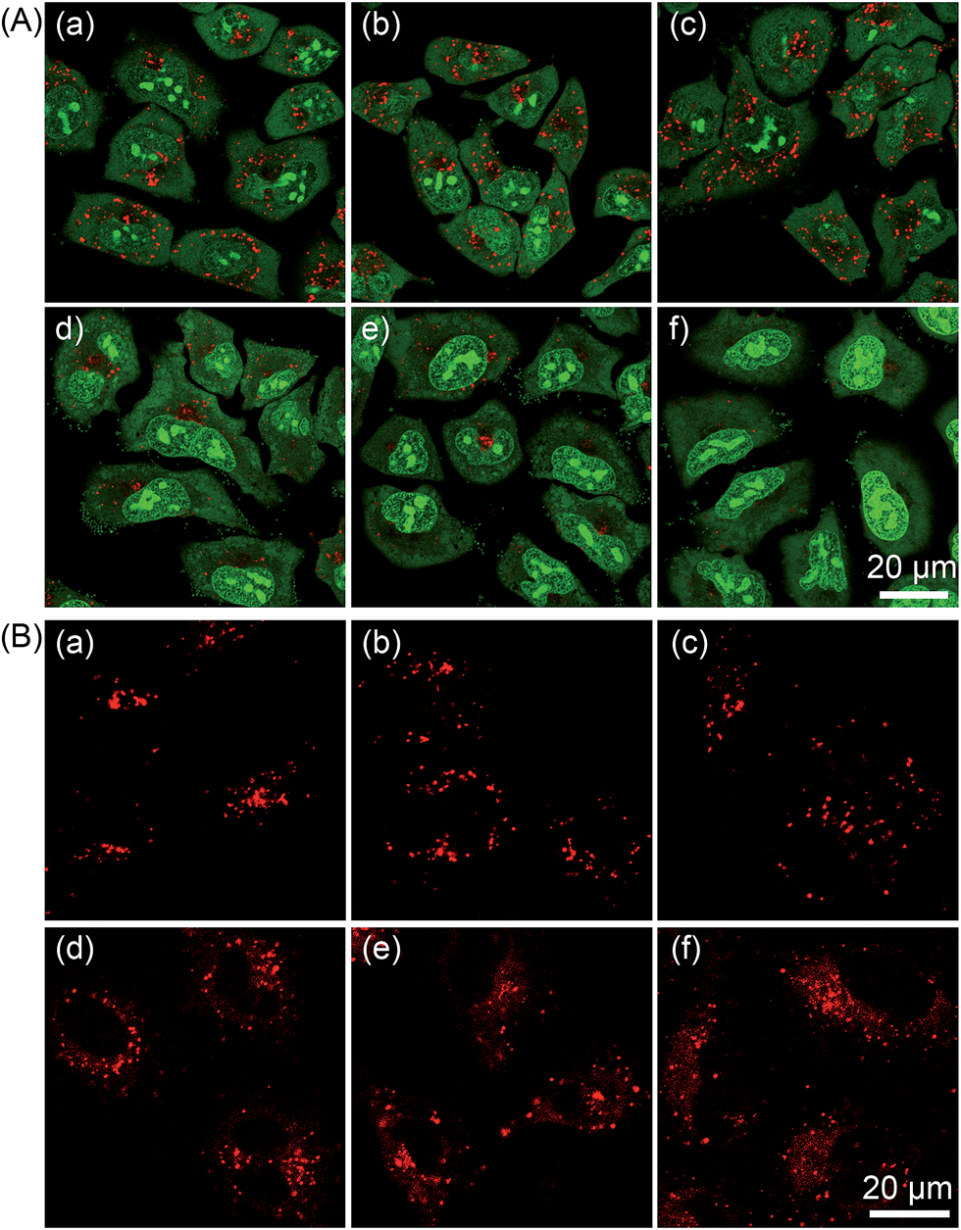
(A) Observation of lysosomal disruption in A549 cells caused by **2**-mediated PDT by AO (5 μM) staining. The cells were treated with (a) vehicle (1% DMSO) in the dark, (b) light alone, (c) **2** (0.5 μM) in the dark, (d) **2** (0.12 μM) with light, (e) **2** (0.25 μM) with light and (f) **2** (0.5 μM) with light. (B) Observation of cathepsin B release from lysosomes to the cytosol as induced by **2**-mediated PDT in A549 cells using the fluorogenic substrate Magic Red MR-(RR)_2_. The cells were treated with (a) vehicle (1% DMSO) in the dark, (b) light alone, (c) **2** (0.5 μM) in the dark, (d) **2** (0.12 μM) with light, (e) **2** (0.25 μM) with light and (f) **2** (0.5 μM) with light. The light dose is 36 J cm^–2^.

It has been reported that PSs localized in the lysosomes can increase production of cellular ROS and lead to uncontrolled lysosomal permeability *via* massive peroxidation of membrane lipids.^30^ The lysosomal integrity of PDT-treated A549 cells was detected by acridine orange (AO) staining. AO is an effective probe to study the integrity of the acidic organelles, because it emits red fluorescence in lysosomes and green fluorescence in the cytosol and nuclei.^10^ As shown in Fig. 9A, A549 cells treated with light or **2** (0.5 μM) alone displayed distinct red fluorescence in lysosomes. However, the red fluorescence of AO remarkably decreased in a PS concentration-dependent manner when the cells were incubated with **2** and irradiated with light, which indicated that lysosomes were damaged upon PDT treatment.

LMP can cause the release of lysosomal proteases, *e.g.*, cathepsin B, from lysosomes to cytosol to promote apoptosis.^23^ The intracellular activity of cathepsin B upon **2**-mediated PDT was detected using the fluorogenic cathepsin B substrate Magic Red MR-(RR)_2_.^31^ The control cells displayed red fluorescence mostly localized in the lysosomes, while PDT-treated cells showed gradually diffused red fluorescence in a PS concentration-dependent manner (Fig. 9B), which indicated the release of cathepsin B from lysosomes to cytosol.

### Selectivity in killing cancer over normal cells

The transformations of lysosomes in cancer cells are numerous as compared with those in normal cells, and most of these changes in the lysosomal compartment in cancer cells are viewed as pro-oncogenic. In this regard, cancer cells display higher susceptibility to lysosomal death pathways.^32^ A co-culture model of A549 and LO2 cells was used to further demonstrate the selectivity of **2**-mediated PDT toward cancer cells over normal cells (Fig. S19†). LO2 cells were distinguished from A549 cells by labeling the nuclei with Hoechst 33342. The control cells irradiated with light alone were undamaged as they were stained by neither annexin V nor propidium iodide. After the co-cultured cells were treated with **2** (0.5 μM) and irradiated with light (36 J cm^–2^), most of A549 cells (Hoechst 33342 negative) were stained by both annexin V and propidium iodide, whereas these phenomena were not found in LO2 cells (Hoechst 33342 positive). These results indicate that **2**-mediated PDT shows high selectivity towards cancer over normal cells.

### Real-time monitoring of lysosomal integrity during PDT

**Fig. 10:**
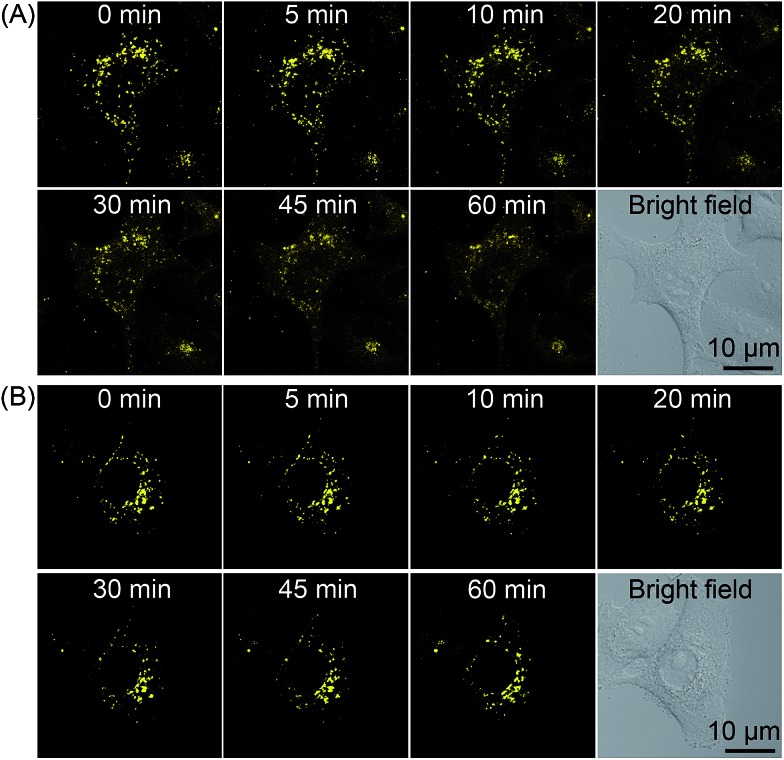
Real-time monitoring of lysosomal integrity in A549 cells loaded with **2** (10 μM, 12 h) (A) with or (B) without PDT treatment (120 s, 4.8 J cm^–2^).

As **2** shows pH-responsive phosphorescence, and it can also act as a lysosome-specific imaging probe and simultaneously induce photodamage to lysosomes, we conceive that it may be utilized to monitor lysosomal integrity during PDT. The changes in the phosphorescence of **2** (10 μM, 12 h) in A549 cells upon PDT treatment (120 s, 4.8 J cm^–2^) were monitored in real-time by confocal microscopy (Fig. 10A and Movie S1†). The images taken immediately after light irradiation showed bright distinct phosphorescence in a punctate pattern, which indicated the integrity of lysosomes. The phosphorescence gradually decreased and became diffused during the next 1 h, which suggested that lysosomal integrity was jeopardized by **2**-mediated PDT. Additionally, the phenomena was not observed in **2**-loaded A549 cells without PDT treatment (Fig. 10B and Movie S2†). The results demonstrate that **2** can realize self-feedback of the lysosomal damage during PDT in real-time, which provides a reliable and convenient method for therapeutic monitoring and treatment assessment.

## Conclusions

In summary, four cyclometalated iridium(iii) complexes with β-carboline ligands have been developed as lysosome-targeted and acidic pH-activatable cell imaging agents and PSs. These complexes show enhanced phosphorescent emission and ^1^O_2_ generation in tumor/lysosome-related acidic environments (pH ≤ 6.5). Complex **2** displays remarkable phototoxicity against cancer cells and high selectivity for cancer cells over normal cells. Mechanism studies show that **2**-mediated PDT mainly induces caspase- and ROS-dependent apoptotic cell death through lysosomal damage and cathepsin B release. Interestingly, complex **2** can be utilized to monitor lysosomal integrity during PDT, which provides a convenient method for *in situ* monitoring of therapeutic effect. However, the practical application of these PSs in PDT may be limited to superficial tumors or other skin diseases due to the short excitation wavelength (425 nm). Stimuli-responsive iridium complexes with longer absorption/emission wavelengths or larger multi-photon absorption cross-sections are likely to be more valuable for the image-guided therapy of deep-seated tumors. Overall, our work provides new insight into the design of multifunctional theranostic phosphorescent metal complexes as smart targeted PDT agents that can enhance tumor selectivity and monitor the therapeutic outcome simultaneously.

## Supplementary Material

Supplementary movie

Supplementary movie

Supplementary information

Crystal structure data

## References

[cit1] Wilbuer A., Vlecken D. H., Schmitz D. J., Kraling K., Harms K., Bagowski C. P., Meggers E. (2010). Angew. Chem., Int. Ed..

[cit2] Lo K. K., Zhang K. Y., Leung S. K., Tang M. C. (2008). Angew. Chem., Int. Ed..

[cit3] Gao R. M., Ho D. G., Hernandez B., Selke M., Murphy D., Djurovich P. I., Thompson M. E. (2002). J. Am. Chem. Soc..

[cit4] Dolmans D. E., Fukumura D., Jain R. K. (2003). Nat. Rev. Cancer.

[cit5] Agostinis P., Berg K., Cengel K. A., Foster T. H., Girotti A. W., Gollnick S. O., Hahn S. M., Hamblin M. R., Juzeniene A., Kessel D., Korbelik M., Moan J., Mroz P., Nowis D., Piette J., Wilson B. C., Golab J. (2011). Ca-Cancer J. Clin..

[cit6] Lovell J. F., Liu T. W., Chen J., Zheng G. (2010). Chem. Rev..

[cit7] Tannock I. F., Rotin D. (1989). Cancer Res..

[cit8] McDonnell S. O., Hall M. J., Allen L. T., Byrne A., Gallagher W. M., O'Shea D. F. (2005). J. Am. Chem. Soc..

[cit9] Han J., Burgess K. (2010). Chem. Rev..

[cit10] Boya P., Kroemer G. (2008). Oncogene.

[cit11] Groth-Pedersen L., Jaattela M. (2013). Cancer Lett..

[cit12] Saftig P., Sandhoff K. (2013). Nature.

[cit13] He L., Tan C. P., Ye R. R., Zhao Y. Z., Liu Y. H., Zhao Q., Ji L. N., Mao Z. W. (2014). Angew. Chem., Int. Ed..

[cit14] Murphy L., Congreve A., Palsson L. O., Williams J. A. G. (2010). Chem. Commun..

[cit15] He L., Liao S. Y., Tan C. P., Ye R. R., Xu Y. W., Zhao M., Ji L. N., Mao Z. W. (2013). Chem.–Eur. J..

[cit16] Zhao N., Wu Y. H., Wen H. M., Zhang X., Chen Z. N. (2009). Organometallics.

[cit17] Lindig B. A., Rodgers M. A. J., Schaap A. P. (1980). J. Am. Chem. Soc..

[cit18] Wessels J. M., Foote C. S., Ford W. E., Rodgers M. A. (1997). Photochem. Photobiol..

[cit19] Nakamaru K. (1982). Bull. Chem. Soc. Jpn..

[cit20] Lo K. K. W., Chan B. T. N., Liu H. W., Zhang K. Y., Li S. P. Y., Tang T. S. M. (2013). Chem. Commun..

[cit21] Knop K., Hoogenboom R., Fischer D., Schubert U. S. (2010). Angew. Chem., Int. Ed..

[cit22] Jahde E., Glusenkamp K. H., Rajewsky M. F. (1991). Cancer Chemother. Pharmacol..

[cit23] Galluzzi L., Bravo-San Pedro J. M., Kroemer G. (2014). Nat. Cell Biol..

[cit24] Vermes I., Haanen C., Reutelingsperger C. (2000). J. Immunol. Methods.

[cit25] Mroz P., Yaroslavsky A., Kharkwal G. B., Hamblin M. R. (2011). Cancers.

[cit26] Taylor R. C., Cullen S. P., Martin S. J. (2008). Nat. Rev. Mol. Cell Biol..

[cit27] Li J., Yuan J. (2008). Oncogene.

[cit28] LeBel C. P., Ischiropoulos H., Bondy S. C. (1992). Chem. Res. Toxicol..

[cit29] Li M. Y., Cline C. S., Koker E. B., Carmichael H. H., Chignell C. F., Bilski P. (2001). Photochem. Photobiol..

[cit30] Buytaert E., Dewaele M., Agostinis P. (2007). Biochim. Biophys. Acta, Rev. Cancer.

[cit31] Mediavilla-Varela M., Pacheco F. J., Almaguel F., Perez J., Sahakian E., Daniels T. R., Leoh L. S., Padilla A., Wall N. R., Lilly M. B., de Leon M., Casiano C. A. (2009). Mol. Cancer.

[cit32] Kirkegaard T., Jaattela M. (2009). Biochim. Biophys. Acta, Mol. Cell Res..

