# Supplementary material for: Cyclometalated iridium(iii) complexes as lysosome-targeted photodynamic anticancer and real-time tracking agents
Source: Chem Sci. 2015 Jul 22;6(10):5409–18. doi: 10.1039/c5sc01955a (PMC5947539; doi:10.1039/c5sc01955a)
Supplement: Supplementary file 3 [file SC-006-C5SC01955A-s003.pdf]

Electronic Supplementary Material (ESI) for Chemical Science.
This journal is © The Royal Society of Chemistry 2015

Electronic Supplementary Information

Cyclometalated iridium(III) complexes as lysosome-targeted photodynamic anticancer and real-time tracking agents

Liang He, Yi Li, Cai-Ping Tan,* Rui-Rong Ye, Mu-He Chen, Jian-Jun Cao, Liang-Nian Ji and Zong-Wan Mao*

Table of Contents

Experimental section……………………S3

Materials and measurements……………………S3

Synthesis and characterization……………………S3

Crystallographic structure determination……………………S5

Measurement of two-photon absorption (TPA) cross-section……………………S6

pH-dependent $^1O_2$ production quantum yields……………………S6

Cell lines and culture conditions……………………S7

PDT activity in vitro……………………S7

Lipophilicity……………………S8

Cellular uptake (ICP-MS)……………………S8

Colocalization assay……………………S8

Cellular uptake mechanism studies……………………S8

pH-dependent emission in A549 cells……………………S9

Annexin V/propidium iodide double staining assay……………………S9

Hoechst 33342 staining……………………S9

Caspase-3/7 activity assay……………………S10

Cellular ROS detection……………..……………………………………………………….S10

AO staining……………………...…………………………………………………….S10

Detection of cathepsin B release………………………………………………………S11

Co-culture of A549 cells and LO2 cells………………………………………………S11

Real-time monitoring of lysosomal integrity upon PDT treatment…………………….…..S11

Statistical analysis……………………..……………………………………………..…..S12

<u>**Supporting figures and tables**</u>……….....……………..………………………………..…..S13

Fig. S1 Chemical structures of ligands **L1** and **L2**……..S13

Fig. S2−S6 ^{1}H NMR spectra of **L2** and complexes **1**−**4**………..……………………..............S13

Fig. S7 X-ray crystal structure of **L2**…………………………………………………….S16

Fig. S8 and S9 UV-Vis absorption spectra of **1**−**4**………………………………...………..S16

Fig. S10 Plots of emission intensity versus pH ………………………………………………S17

Fig. S11 In vitro PDT dose-response curves for **1**−**4**…………………………………………S18

Fig. S12−S14 Colocalization assay……………………………………….....……………S19

Fig. S15 and S16 Cellular uptake mechanisms…………………….……....………………S22

Fig. S17 Annexin V/propidium iodide double staining (confocal)…………….…………….…..S24

Fig. S18 Hoechst 33342 staining.……………………….………………………..…………S25

Fig. S19 Selective induction of apoptosis in the A549/LO2 co-culture model……….………S26

Table S1 Crystallographic data of **L2** and **4**…………………...…....……………………..S27

Table S2 Selected bond lengths and bond angles of **L2** and **4**……………...............……......S28

Table S3 PDT activities against MCF-7 cells……...…………..S29

Table S4 Lipophilicity and cellular uptake efficiency of complexes **1**−**4**………………...…S30

<u>**Supporting references**</u>…….……………………………………….……………..…….S31

<u>**Movies**</u>…..…………………………………………………….……..……….………S31

Experimental section

Materials and measurements

Iridium chloride hydrate (Alfa Aesar, USA), ppy (2-phenylpyridine, Sigma Aldrich, USA), dfppy (2-(2,4-difluorophenyl)pyridine, Sigma Aldrich, USA), benzimidazole-2-carbaldehyde (J&K Scientific Ltd., China), tryptamine (Alfa Aesar, USA), NH_4PF_6 (Alfa Aesar, USA), cisplatin (Sigma Aldrich, USA), DMSO (Sigma Aldrich, USA), MTT (3-(4,5-dimethylthiazol-2-yl)-2,5-diphenyltetrazoli-um bromide, Sigma Aldrich, USA), disodium hydrogen phosphate (Sigma Aldrich, USA), citric acid (Sigma Aldrich, USA), ABDA (Sigma Aldrich, USA), $[Ru(bpy)_3]Cl_2$ (Sigma Aldrich, USA), LTDR (Life Technologies, USA), MTDR (Life Technologies, USA), Hoechst 33342 (Sigma Aldrich, USA), propidium iodide (Sigma Aldrich, USA), PBS (phosphate buffered saline, Sigma Aldrich, USA), CCCP (Sigma Aldrich, USA), nigericin (Cayman Chemical, USA), z-VAD-fmk (Sigma Aldrich, USA), NaN_3 (Sigma Aldrich, USA), AO (Sigma Aldrich, USA), DCF-DA (Sigma Aldrich, USA) and rhodamine 6G (Sigma Aldrich, USA) were used as received. Annexin V-FITC apoptosis detection kit was purchased from Sigma Aldrich (USA). Caspase-3/7 activity kit was purchased from Promega (USA). Magic Red MR-$(RR)_2$ was purchased from Immunochemistry Tech (USA). The iridium(III) complexes were proven to be stable for at least 48 h at room temperature in PBS as monitored by UV-visible spectroscopy. All the compounds tested were dissolved in DMSO just before the experiments, and the final concentration of DMSO was kept at 1% (v/v). NMR spectra were recorded on a Bruker Avance 400 spectrometer. Shifts were referenced relative to the internal solvent signals. Microanalysis (C, H, and N) was carried out using an Elemental Vario EL CHNS analyzer (Germany). UV-Vis absorption spectra were recorded on a Varian Cary 300 spectrophotometer (USA). Steady-state emission spectra and lifetime measurements were performed on an Edinburgh FLS 920 Spectrometer (UK). ESI-MS spectra were recorded on a Thermo Finnigan LCQ DECA XP spectrometer (USA). The quoted m/z values represented the major peaks in the isotopic distribution.

Synthesis and characterization

Ligand **L1** was synthesized as previously reported.[1] The dimeric iridium(III) precursors ($Ir_2(ppy)_4Cl_2$ and $Ir_2(dfppy)_4Cl_2$) were prepared according to the literature method.[2]

1-(2-benzimidazolyl)-β-carboline (L2). Ligand **L2** was synthesized in a manner identical to that described previously for **L1**.[1] Briefly, a mixture of benzimidazole-2-carbaldehyde (0.731 g, 5.00 mmol) and tryptamine (0.810 g, 5.00 mmol) in dry anisole (200 mL) was heated to 155 ^{o}C over a period of 2 h and then 10% Pd/C (1.20 g) was added and refluxed for 22 h. The reaction mixture was filtered while hot, and rotary evaporation of the filtrate gave dark yellow oil, which was then dissolved in ethanol (20 mL). The crude product was purified by recrystallization from ethanol and pure compound was obtained by filtration. Yield: 1.07 g (75%). ^{1}H NMR (400 MHz, d_6-DMSO): δ = 13.289 (s, 1H), 11.663 (s, 1H), 8.514 (d, J = 5.1 Hz, 1H), 8.269 (t, J = 7.4 Hz, 2H), 7.979 (d, J = 8.2 Hz, 1H), 7.886–7.865 (m, 1H), 7.611–7.569 (m, 2H), 7.303–7.266 (m, 3H). ESI-MS (CH_3OH): m/z calcd for $[M+H]^+$, 285.31; found: 285.4. Elemental analysis calcd (%) for $C_{18}H_{12}N_4$: C, 76.04; H, 4.25; N, 19.71; found: C, 76.00; H, 4.32; N, 19.57.

General method for [Ir(N^C)$_2$(N^N)](PF$_6$). A mixture of $Ir_2(N\text{^}C)_4Cl_2$ (0.4 mmol, 1 equiv.) and **L1/L2** (0.8 mmol, 2 equiv.) in CH_2Cl_2/CH_3OH (2:1, v/v) was heated to reflux under an inert atmosphere of nitrogen in the dark. After 4 h, the solution was cooled to room temperature and then a 6-fold excess of NH_4PF_6 was added, and the mixture was stirred for another 1 h. The mixture was filtered and the filtrate was evaporated to dryness under reduced pressure. The solid obtained was dissolved in CH_2Cl_2 and purified by column chromatography on silica gel eluted with CH_2Cl_2/acetone (10:1, v/v). The desired product was purified by recrystallization from CH_2Cl_2/diethyl ether or acetone/*n*-hexane.

[Ir(ppy)$_2$(L1)](PF$_6$) (1). Complex **1** was obtained as a yellow powder. Yield: 0.514 g (73%). ^{1}H NMR (400 MHz, d_6-DMSO): δ = 12.641 (s, 1H), 12.082 (s, 1H), 8.213–8.164 (m, 3H), 8.051 (d, J = 5.8 Hz, 1H), 7.903–7.805 (m, 5H), 7.631 (dd, J = 14.8, 5.9 Hz, 3H), 7.563 (s, 1H), 7.488 (d, J = 5.8 Hz, 1H), 7.326 (t, J = 7.5 Hz, 1H), 7.127 (t, J = 6.4 Hz, 1H), 7.064 (t, J = 6.6 Hz, 1H), 6.997 (t, J = 7.2 Hz, 1H), 6.922 (dt, J = 14.7, 7.4 Hz, 2H), 6.828 (t, J = 7.4 Hz, 1H), 6.522 (s, 1H), 6.360 (d, J = 7.2 Hz, 1H), 6.291 (d, J = 7.3 Hz, 1H). ESI-MS (CH_2Cl_2): m/z calcd for $[M-PF_6]^+$, 735.18; found: 735.2. Elemental analysis calcd (%) for $C_{36}H_{26}N_6PF_6Ir$: C, 49.15; H, 2.98; N, 9.55; found: C, 49.32; H, 3.08; N, 9.59.

[Ir(ppy)$_2$(L2)](PF$_6$) (2). Complex **2** was obtained as an orange powder. Yield: 0.595 g (80%). ^{1}H NMR (400 MHz, d_6-DMSO): δ = 11.984 (s, 1H), 10.615 (s, 1H), 8.224–8.129 (m, 3H), 8.089–8.050 (m, 2H), 7.893 ((t, J = 8.2 Hz, 2H), 7.801–7.705 (m, 3H), 7.649–7.585 (m, 3H), 7.510 (d, J = 5.8 Hz, 1H), 7.320 (t, J = 7.5 Hz, 1H), 7.046–6.873 (m, 7H), 6.652 (t, J = 7.5 Hz, 1H), 6.405 (t, J = 8.4 Hz, 2H), 6.104 (d, J = 8.1 Hz, 1H). ESI-MS (CH_2Cl_2): m/z calcd for $[M-PF_6]^+$, 785.20; found: 785.1. Elemental analysis calcd (%) for $C_{40}H_{28}N_6PF_6Ir$: C, 51.67; H, 3.04; N, 9.04; found: C, 51.89; H, 3.13; N, 9.10.

[Ir(dfppy)$_2$(L1)](PF$_6$) (3). Complex **3** was obtained as a green-yellow powder. Yield: 0.518 g (68%). ^{1}H NMR (400 MHz, d_6-DMSO): δ = 14.068 (s, 1H), 12.058 (s, 1H), 8.236 (d, J = 8.0 Hz, 3H), 8.124 (s, 1H), 7.967–7.924 (m, 2H), 7.867 (d, J = 8.2 Hz, 1H), 7.648–7.487 (m, 5H), 7.336 (t, J = 7.3 Hz, 1H), 7.216 (d, J = 6.6 Hz, 1H), 7.148 (t, J = 6.5 Hz, 1H), 6.920–6.820 (m, 2H), 6.676–6.567 (m, 1H), 5.753 (d, J = 8.1 Hz, 1H), 5.672 (d, J = 8.2 Hz, 1H). ESI-MS (CH_2Cl_2): m/z calcd for $[M-PF_6]^+$, 807.15; found: 807.3. Elemental analysis calcd (%) for $C_{36}H_{22}N_6PF_{10}Ir$: C, 45.43; H, 2.33; N, 8.83; found: C, 45.53; H,2.35; N, 8.89.

[Ir(dfppy)$_2$(L2)](PF$_6$) (4). Complex **4** was obtained as an orange powder. Yield: 0.625 g (78%). ^{1}H NMR (400 MHz, d_6-DMSO): δ = 14.341 (s, 1H), 12.457 (s, 1H), 8.465 (d, J = 5.6 Hz, 1H), 8.377 (d, J = 8.0 Hz, 1H), 8.308 (d, J = 8.0 Hz, 1H), 8.341 (d, J = 8.4 Hz, 1H), 7.946 (dt, J = 14.1, 6.9 Hz, 4H), 7.821–7.782 (m, 2H), 7.738 (d, J = 5.6 Hz, 1H), 7.660 (d, J = 5.6 Hz, 1H), 7.465 (t, J = 7.5 Hz, 2H), 7.209–7.135 (m, 3H), 7.028 (dt, J = 21.9, 11.0 Hz, 2H), 6.298 (d, J = 8.3 Hz, 1H), 5.813 (dd, J = 8.4, 2.0 Hz, 1H), 5.717 (dd, J = 8.3, 2.0 Hz, 1H). ESI-MS (CH_2Cl_2): m/z calcd for $[M-PF_6]^+$, 857.16; found: 857.3. Elemental analysis calcd (%) for $C_{40}H_{24}N_6PF_{10}Ir$: C, 47.95; H, 2.41; N, 8.39; found: C, 48.11; H, 2.50; N, 8.42.

Crystallographic structure determination

Crystals of (**L2**)$_2$•$(CH_3)_2CO$ and **4**•$2CH_3CH_2OH$ suitable for X-ray analysis were obtained by slow evaporation of the acetone-methanol mixture solution of **L2** and the acetone-ethanol mixture solution of **4**, respectively. X-ray diffraction measurements were performed on a Bruker Smart 1000 CCD diffractometer with Mo Kα radiation (λ = 0.71073 Å) at 173 K or 150 K. The crystal structures were

solved by direct methods with program SHELXS and refined using the full-matrix least-squares program SHELXL.[3] The CCDC deposit numbers, crystallographic data, details of data collection and structure refinements are listed in Table S1. Selected bond distances and angles are listed in Table S2. The structural plots were drawn using the xp package in SHELXTL at a 50% thermal ellipsoids probability level.

Measurement of two-photon absorption (TPA) cross-section

The TPA cross-sections were measured as previously reported.[4] Briefly, the two-photon excited fluorescence (TPEF) spetra were acquired with a nanosecond pulsed laser (OpoletteTM 355II; pulse width ≤ 100 fs; 80 MHz repetition rate; Spectra Physics Inc., USA). The two-photon induced fluorescence intensity was measured at 730−970 nm by using rhodamine 6G as the reference.[5] The intensities of TPEF of the reference and samples emitted at the same excitation wavelength were determined. The TPA cross sections were calculated according to the following equation.[6]

$$\delta_s = \delta_r \frac{\Phi_r c_r I_s n_s}{\Phi_s c_s I_r n_r}$$

Where I is the integrated fluorescence intensity, c is the concentration, n is the refractive index, Φ is the quantum yield, subscript 'r' stands for reference samples, and 's' stands for the samples.

pH-dependent $^1O_2$ production quantum yields

The quantum yields for $^1O_2$ production (Φ_Δ) of the complexes under irradiation in aerated disodium hydrogen phosphate/citric acid buffer solutions (pH 3.0, 5.0. 6.5 and 7.4) were evaluated using a steady-state method with ABDA as the $^1O_2$ indicator[7] and $[Ru(bpy)_3]Cl_2$ as the standard (Φ_Δ = 0.18 in H_2O).[8] Briefly, air-equilibrated buffer solutions containing the tested complexes and ABDA (100 μM) were prepared in the dark and irradiated with a 425 nm LED light array. The absorption maxima of ABDA (377, 378, 380 and 380 nm at pH 3.0, 5.0, 6.5 and 7.4, respectively) were recorded every 20 s. The absorbance at 425 nm of the iridium(III) complexes and $[Ru(bpy)_3]Cl_2$ was kept at 0.15. The Φ_Δ of the iridium(III) complexes were calculated according to the following equation.

$$\Phi_{\Delta(x)} = \Phi_{\Delta(std)} \times (\frac{S_x}{S_{std}}) \times (\frac{F_{std}}{F_x})$$

where subscripts x and std designate the sample and $[Ru(bpy)_3]Cl_2$, respectively, S stands for the

slope of plot of the absorption maxima of ABDA against the irradiation time (s). *F* stands for the absorption correction factor, which is given by $F = 1 - 10^{-OD}$ (OD represents the optical density of sample and $[Ru(bpy)_3]Cl_2$ at 425 nm).

Cell lines and culture conditions

A549, A549cisR, MCF-7 and LO2 cells were obtained from Experimental Animal Center of Sun Yat-sen University (Guangzhou, China). Cells were maintained in DMEM (Dulbecco's modified Eagle's medium, Gibco BRL) or RPMI 1640 (Roswell Park Memorial Institute 1640, Gibco BRL) medium, which contained 10% FBS (fetal bovine serum, Gibco BRL), 100 μg/mL streptomycin (Gibco BRL), and 100 U/mL penicillin (Gibco BRL). The cells were cultured in a humidified incubator, which provided an atmosphere of 5% CO_2 and 95% air at a constant temperature of 37 °C. In each experiment, cells treated with vehicle control (1% DMSO) were kept as the reference group.

PDT activity in vitro

The cytotoxicity in the dark and phototoxicity of the tested compounds toward A549, A549cisR, MCF-7 and LO2 cell lines was determined by MTT assay.

For **cytotoxicity in the dark**, cells cultured in 96-well plates were grown to confluence. The compounds were dissolved in DMSO (1%, v/v), and diluted with fresh media immediately. the cells were incubated with a series of concentrations of the tested compounds for 44 h at 37 °C. 20 μL of MTT solution was then added to each well, and the plates were incubated for an additional 4 h. The media was carefully removed, and DMSO was added (150 μL per well) and incubated for 10 min with shaking. The absorbance at 595 nm was measured using a microplate reader (Infinite M200 Pro, Tecan, Männedorf, Switzerland).

For **phototoxicity**, the cells were incubated with the tested compounds for 12 h. The media containing the iridium complexes was removed and fresh media without the complexes was added. Then the cells were irradiated with a 425 nm LED light array (40 mW cm^{-2}) for 15 min (36 J cm^{-2}). After another 32 h of incubation, MTT was added. The cytotoxicity was determined as described above. The light dose used was determined based on these considerations: an optimal photocytotoxicity in iridium(III)-treated cells was achieved and no statistical difference in viability was observed between cells kept in the dark and cells irradiated.

Lipophilicity

The lipophilicity of the iridium(III) complexes, which was presented as log $P_{o/w}$ values, was determined according to a reported procedure.[9] Log $P_{o/w}$ is defined as the logarithmic ratio of Ir(III) concentration in *n*-octanol to that in the aqueous phase.

Cellular uptake (ICP-MS)

A549 cells were seeded in 10 cm tissue culture dishes and incubated for 24 h. The media was removed and replaced with fresh media containing the tested complexes (20 μM). After 5 or 12 h incubation, the cells were washed with PBS, trypsinized and collected. The cells were counted, and digested with HNO_3 (65%, 0.2 mL) at room temperature for 24 h. The solution was then diluted to a final volume of 10 mL with Milli-Q water. The concentration of iridium was measured using the XSERIES 2 ICP-MS.

Colocalization assay

A549 cells were incubated with iridium(III) complexes (20 μM) at 37 °C for 4.5 h and further co-incubated with LTDR (150 nM) or MTDR (150 nM) at 37 °C for another 0.5 h. Cells were washed three times with PBS and visualized by confocal microscopy (LSM 710, Carl Zeiss, Göttingen, Germany) with a 63× oil-immersion objective lens immmediately. The wavelengths for one- and two-photon excitation of iridium(III) complexes were 405 nm and 810 nm, respectively. The exicitaiton wavelength of LTDR and LTDR was 633 nm. Emission was collected at 550 ± 30 nm (**1**), 580 ± 30 nm (**2**), 530 ± 30 nm (**3**), 570 ± 30 nm (**4**), 668 ± 20 nm (LTDR) and 665 ± 20 nm (MTDR).

Cellular uptake mechanism studies

A549 cells were incubated with **2** (20 μM) at 4, 25 and 37 °C for 2 h, or pretreated with 20 μM CCCP for 1 h at 37 °C and incubated with **2** (20 μM) at 37 °C for another 2 h. For confocal microscopy analysis, the cells were then washed three times with PBS and visualized by confocal microscopy (LSM 710, Carl Zeiss, Göttingen, Germany). Emission was collected at 580 ± 30 nm upon excitation at 405 nm. For flow cytometry analysis, the cells were washed twice with serum-free RPMI 1640, and the emission intensity of the cells was measured by flow cytometry

(FACSCalibur™, Becton Dickinson, NJ, USA). Data were analyzed by FlowJo software (Tree Star, OR, USA). Ten thousand events were acquired for each sample.

pH-dependent emission in A549 cells

A549 cells were incubated with **2** (20 μM) for 5 h at 37 °C. The media was removed and the cells were then incubated with nigericin (20 μM) in disodium hydrogen phosphate/citric acid buffer solutions (pH 5.0, 6.5 and 7.4) for 10 min. The cells were visualized immmediately by confocal microscopy (LSM 710, Carl Zeiss, Göttingen, Germany). Emission was collected at 580 ± 30 nm upon excitation at 405 nm. The emission intensity (n = 10 cells) was quantified using ImageJ software.

Annexin V/propidium iodide double staining assay

The assay was performed according to the manufacturer's (Sigma Aldrich, USA) protocol. A549 cells were exposed to complex **2** at the indicated concentrations for 24 h with or without light irradiation. After A549 cells were incubated with **2** (0.12, 0.25 or 0.5 μM) for 12 h, the cells were irradiated with a 425 nm LED light array (40 mW cm^{-2}) at different light doses (12, 24 or 36 J cm^{-2}). When necessary, the cells were incubated with NaN_3 (10 mM) for 1 h before PDT treatment. For flow cytometry analysis, the cells were harvested and re-suspended in 500 μL annexin-binding buffer (10 mM HEPES, 140 mM NaCl, 2.5 mM $CaCl_2$, pH 7.4). The cell suspension was stained with 5 μL annexin V and 10 μL propidium iodide at room temperature for 15 min in the dark, and then analyzed immediately by flow cytometry (FACSCalibur™, Becton Dickinson, NJ, USA). The resulting dot blots were analyzed by FlowJo Software (Tree Star, OR, USA). For confocal microscopy analysis, the cells were washed twice with PBS, and then incubated with 500 μL annexin-binding buffer supplemented with 5 μL annexin V and 10 μL propidium iodide at room temperature for 15 min in the dark. The samples were visualized immediately by confocal microscopy (LSM 710, Carl Zeiss, Göttingen, Germany). Emission was collected at 530 ± 20 nm (annexin V) and 620 ± 20 nm (propidium iodide) upon excitation at 488 nm.

Hoechst 33342 staining

A549 cells seeded into 35 mm dishes (Corning) were exposed to complex **2** at the indicated

concentrations for 24 h with or without light irradiation. After A549 cells were incubated with **2** for 12 h, the cells were irradiated with a 425 nm LED light array (40 mW cm^{-2}) for 15 min (36 J cm^{-2}). When necessary, the cells were incubated with NaN_3 (10 mM) for 1 h before PDT treatment. The cells were washed twice with PBS gently and fixed with 4% paraformalclehyde for 30 min at room temprature. The cells were washed with PBS twice, and then Hoechst 33342 (5 μg/mL) in PBS was added to the media by gently shaking in the dark for 10 min. The cells were washed with PBS twice and visualized by confocal microscopy (LSM 710, Carl Zeiss, Göttingen, Germany). Emission was collected at 460 ± 20 nm upon excitation at 405 nm.

Caspase-3/7 activity assay

Caspase-3/7 activity was measured using Caspase-Glo® Assay kit (Promega, Madison, WI, USA) according to the manufacturer's instructions. Briefly, cells were cultured in 96-well plates and treated with complex **2** or cisplatin for 24 h with or without light irradiation. After A549 cells were incubated with **2** or cisplatin for 12 h, the cells were irradiated with a 425 nm LED light array (40 mW cm^{-2}) for 15 min (36 J cm^{-2}). 100 μL of Caspase-Glo® 3/7 reagent was added to each well containing 100 μL culture media. The mixture was incubated at room temperature for 1 h and then the luminescence was measured using a microplate reader (Infinite M200 Pro, Tecan, Männedorf, Switzerland).

Cellular ROS detection

A549 cells seeded into 6-well tissue culture plates (Corning) were treated with complex **2** for 12 h. The cells were further treated with 10 μM DCF-DA for 20 min at 37 °C in the dark and then then irradiated with a 425 nm LED light array (40 mW cm^{-2}) for 15 min (36 J cm^{-2}). After the treatment, the cells were harvested and washed twice with serum-free RPMI 1640, the fluorescence intensity of DCF in A549 cells was measured by flow cytometry (FACSCalibur™, Becton Dickinson, Franklin Lakes, NJ, USA). The results were analyzed by FlowJo Software (Tree Star, OR, USA).

AO staining

A549 cells seeded into 35 mm dishes (Corning) were exposed to complex **2** at the indicated concentrations for 12 h and then irradiated with a 425 nm LED light array (40 mW cm^{-2}) for 15 min

(36 J cm^{-2}). The cells were then washed twice with PBS and incubated with AO (5 μM) at 37 °C for 15 min. The cells were washed twice with PBS and visualized by confocal microscopy (LSM 710, Carl Zeiss, Göttingen, Germany). Emission was collected at 510 ± 20 nm (green) and 625 ± 20 nm (red) upon excitation at 488 nm.

Detection of cathepsin B release

Cathepsin B activity was detected using the fluorogenic susbtrate Magic Red MR-$(RR)_2$ (Immunochemistry Tech, Bloomington, USA) according to the manufacturer's instructions. Briefly, A549 cells seeded into 35 mm dishes (Corning) were exposed to complex **2** at the indicated concentrations for 12 h and then irradiated with a 425 nm LED light array (40 mW cm^{-2}) for 15 min (36 J cm^{-2}). The media was removed and the cells were washed twice with PBS and then incubated with cathepsin B substrate at 37 °C for 1 h. The media was removed and the cells were washed twice with PBS and visualized by confocal microscopy (LSM 710, Carl Zeiss, Göttingen, Germany). Emission was collected at 630 ± 20 nm upon excitation at 543 nm.

Co-culture of A549 cells and LO2 cells

LO2 cells were pre-incubated with Hoechst 33342 at 2.0 μg/mL for 10 min. After being washed with PBS three times, the Hoechst labeled LO2 cells were suspended in fresh media and then seeded into 35 mm dishes (Corning) with similar amount of A549 cells, and incubated for 24 h for recovery. The cell mixtures were exposed to complex **2** (0.5 μM) for 12 h and then irradiated with a 425 nm LED light array (40 mW cm^{-2}) for 15 min (36 J cm^{-2}). The cell mixtures were incubated at 37 °C for another 12 h and then stained with annexin V and propidium iodide as described above in the annexin V/propidium iodide double staining assay section.

Real-time monitoring of lysosomal integrity upon PDT treatment

A549 cells seeded into 35 mm dishes (Corning) were exposed to complex **2** (10 μM) for 12 h and then irradiated with light for 120 s (425 nm, 4.8 J cm^{-2}). The cells were incubated in a humidified incubator, which provided an atmosphere of 5% CO_2 and 95% air at a constant temperature of 37 °C and was connected with the confocal microscope. The cells were immediately visualized after PDT treatment by confocal microscopy (LSM 710, Carl Zeiss, Göttingen, Germany). Photographs were

taken every 5 min. Emission was collected at 580 ± 30 nm upon excitation at 405 nm.

Statistical analysis

All biological experiments were performed at least twice with triplicates in each experiment. Representative results were depicted in this report and data were presented as means ± standard deviations (SD).

Supporting figures and tables

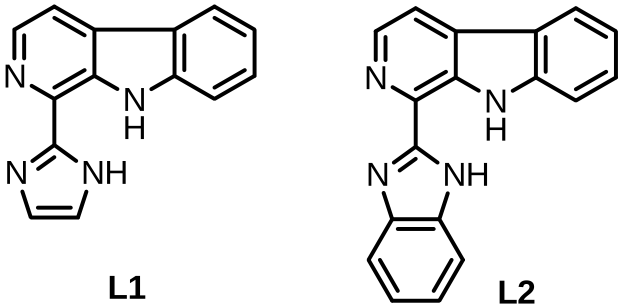

Fig. S1 Chemical structures of ligands **L1** and **L2**.

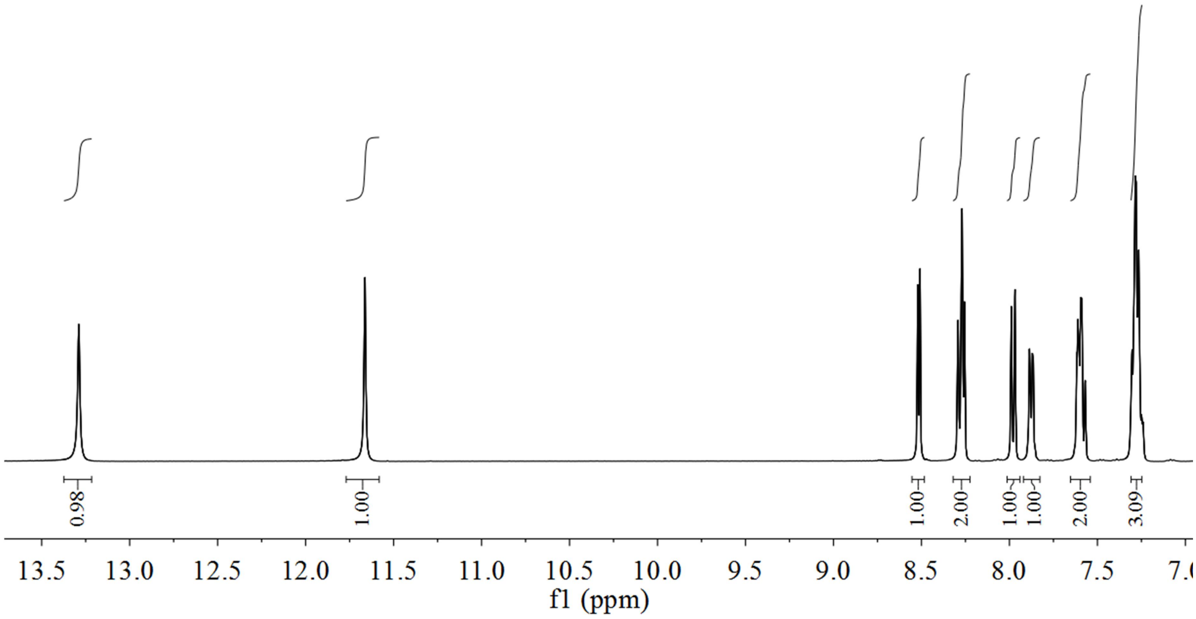

Fig. S2 ^{1}H NMR spectrum of **L2**.

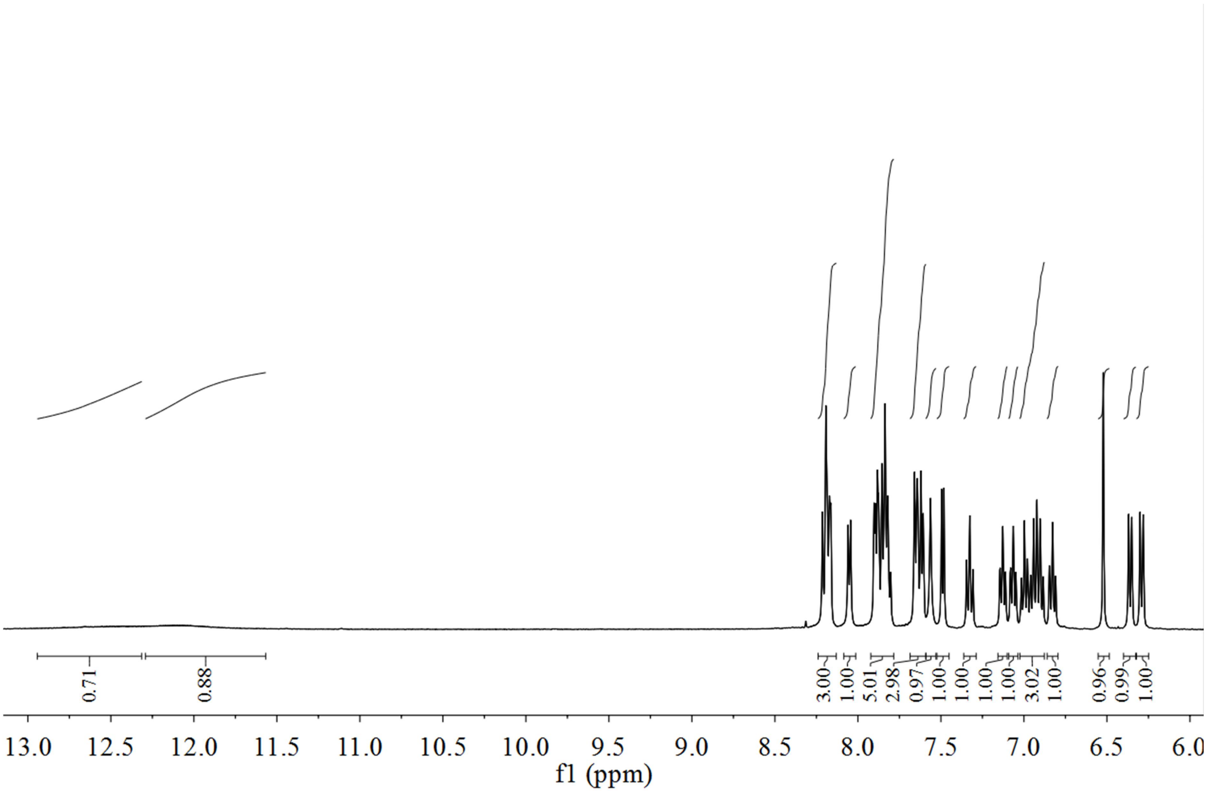

Fig. S3 ¹H NMR spectrum of **1**.

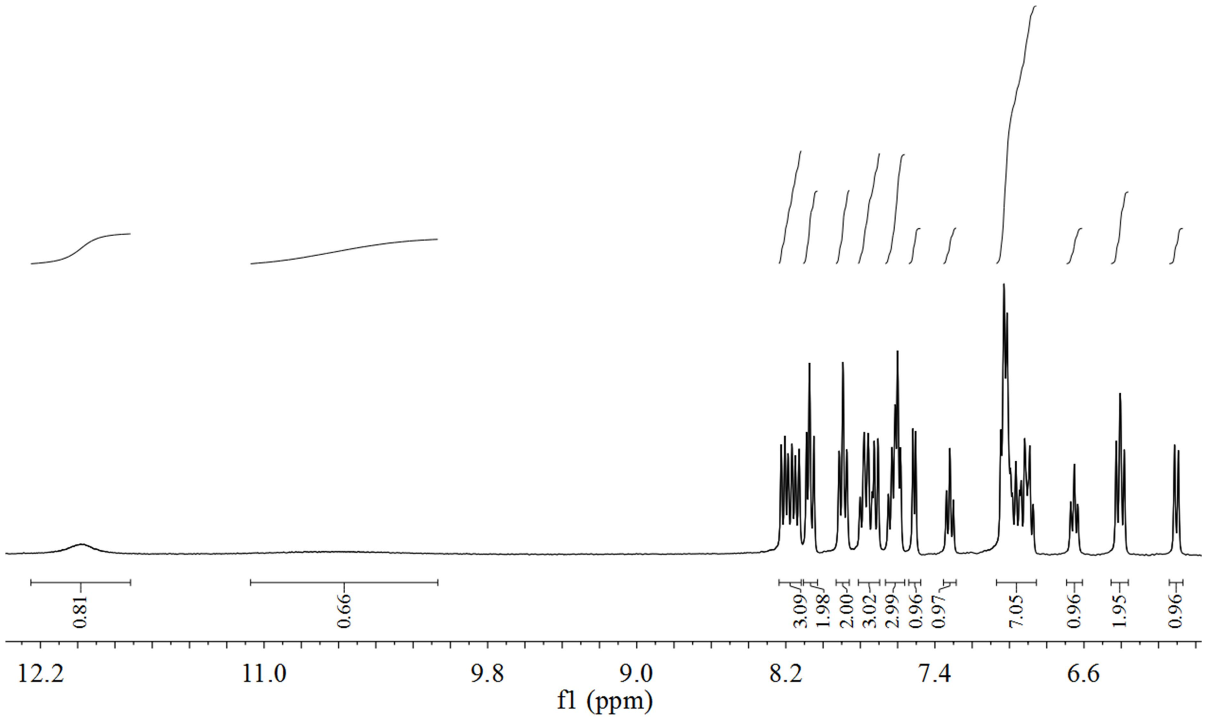

Fig. S4 ¹H NMR spectrum of **2**.

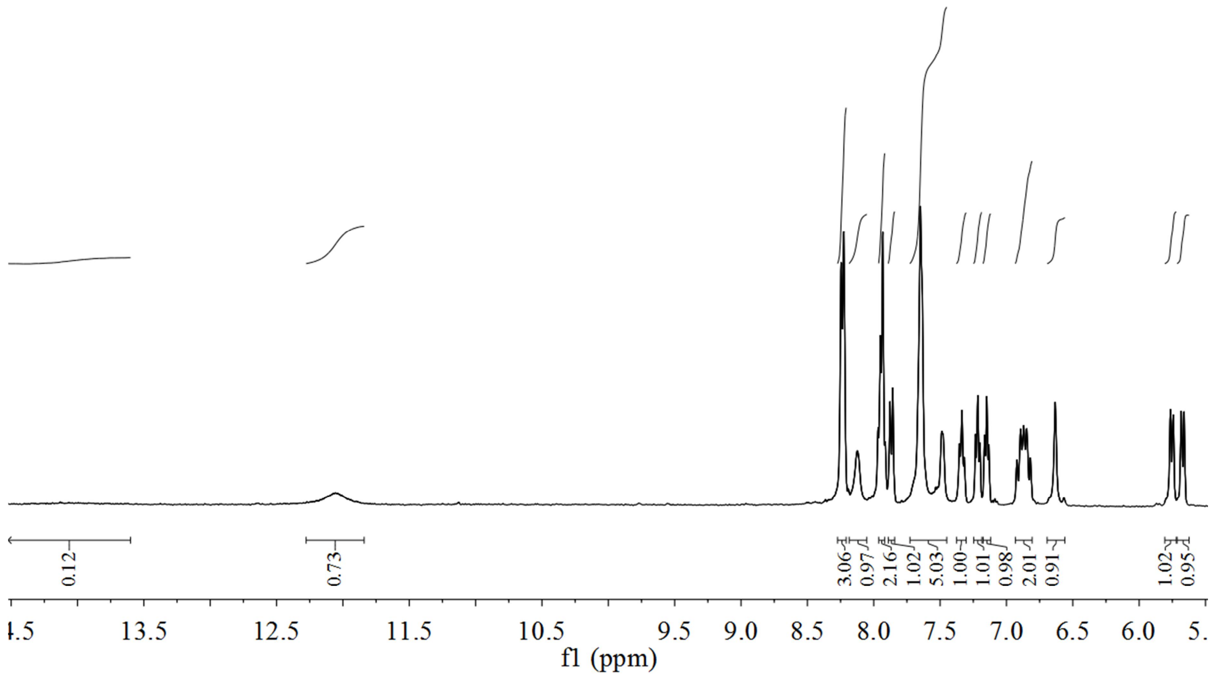

Fig. S5 ^{1}H NMR spectrum of **3**.

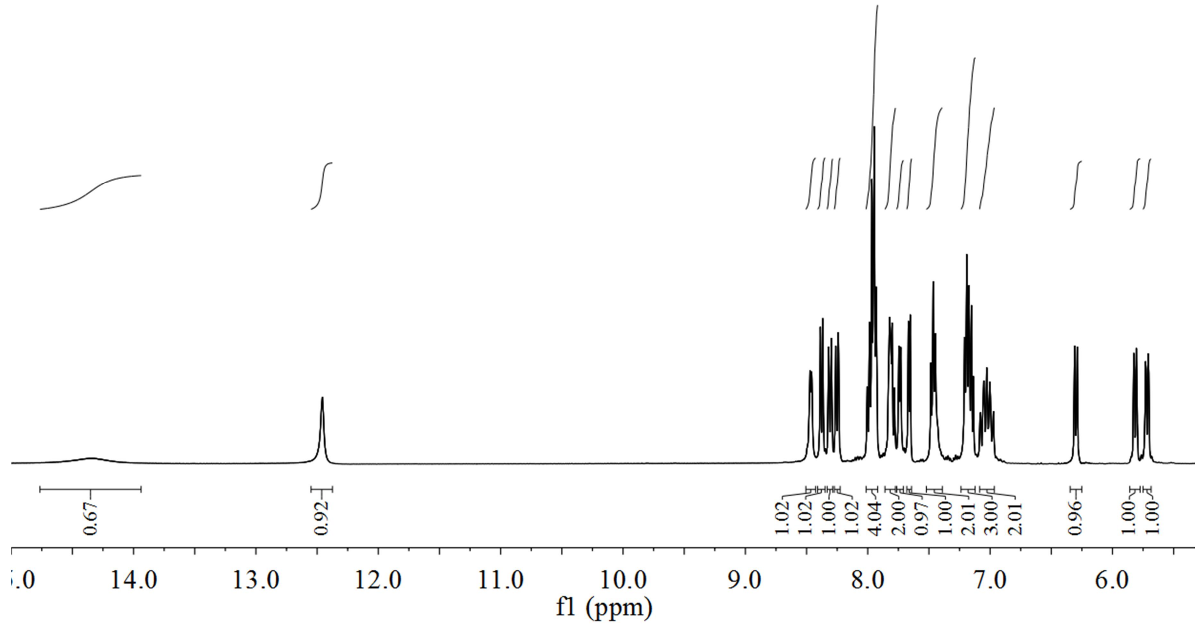

Fig. S6 ^{1}H NMR spectrum of **4**.

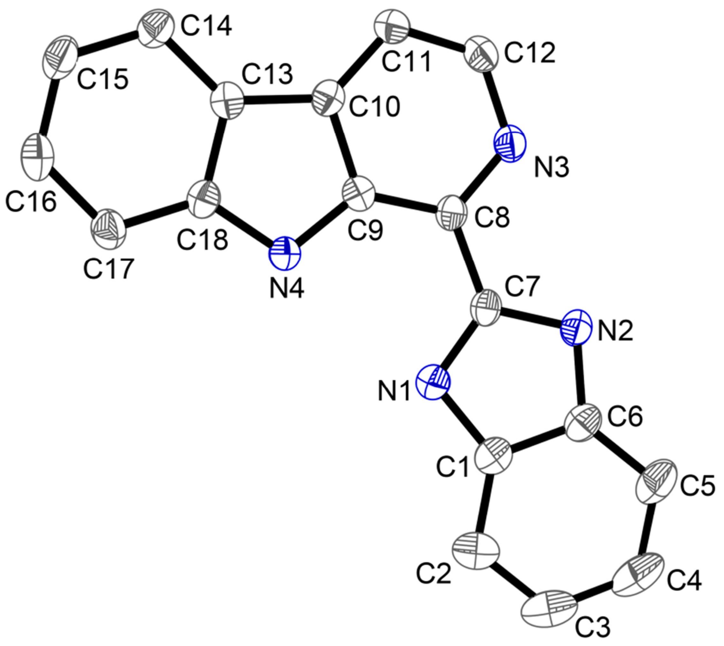

Fig. S7 X-ray crystal structure of $(\mathbf{L2})_2 \bullet (CH_3)_2CO$ with thermal ellipsoids set at the 50% probability level. Only one of two molecules in the asymmetric unit is shown. The H atoms and solvent have been omitted for clarity.

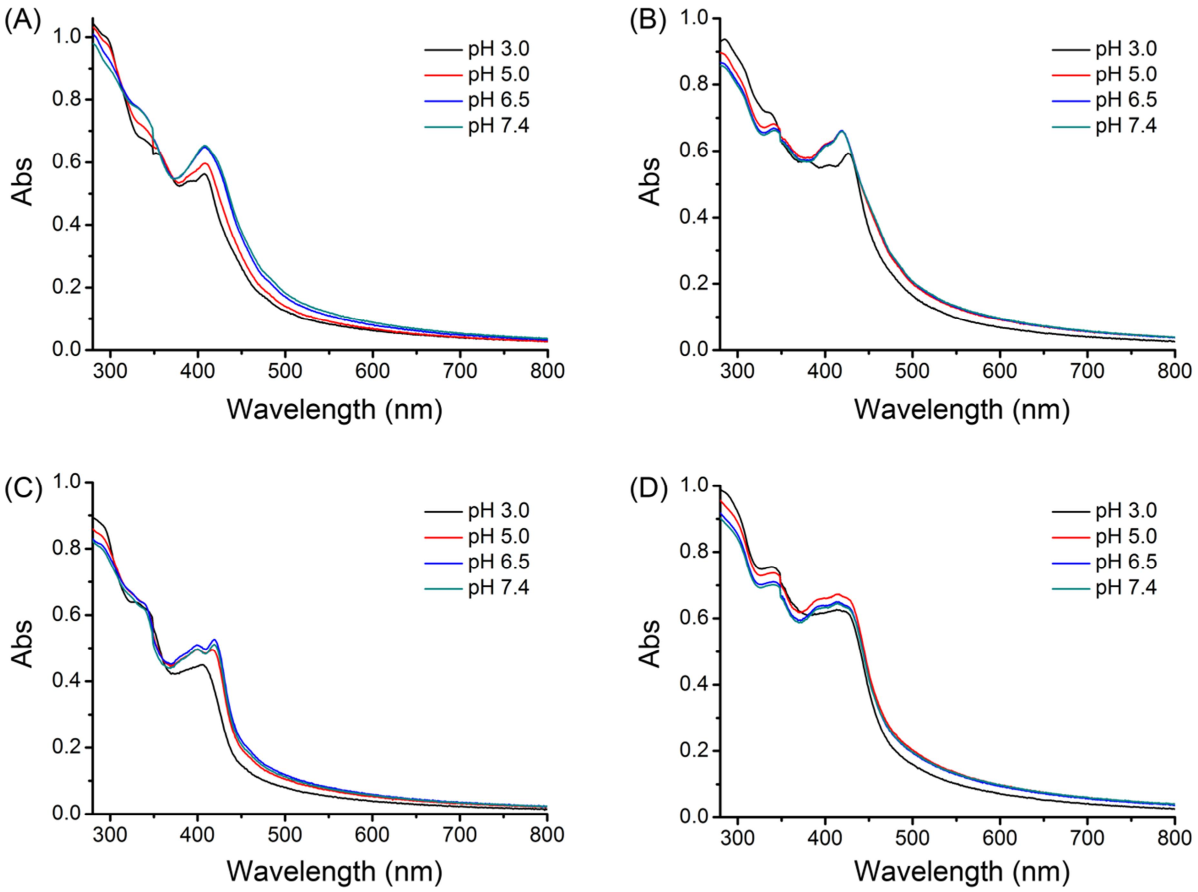

Fig. S8 UV-Vis absorption spectra of (A) **1**, (B) **2**, (C) **3** and (D) **4** (2×10^{-5} μM) measured at pH 3.0, 5.0, 6.5 and 7.4 in disodium hydrogen phosphate/citric acid buffer solutions.

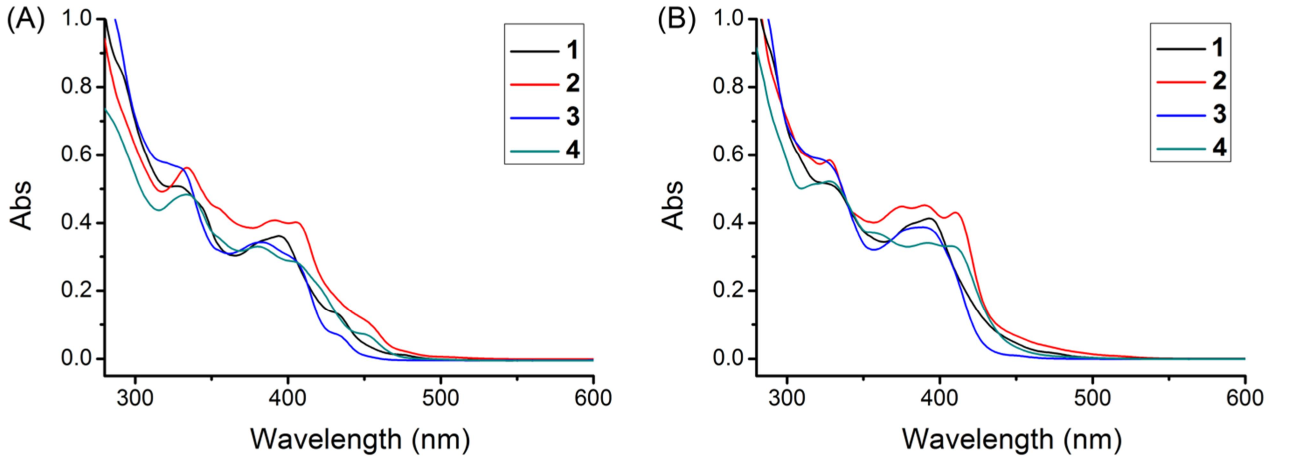

Fig. S9 UV-Vis absorption spectra of iridium(III) complexes **1**–**4** (2×10^{-5} μM) measured in (A) CH_3OH and (B) CH_3CN at 298 K.

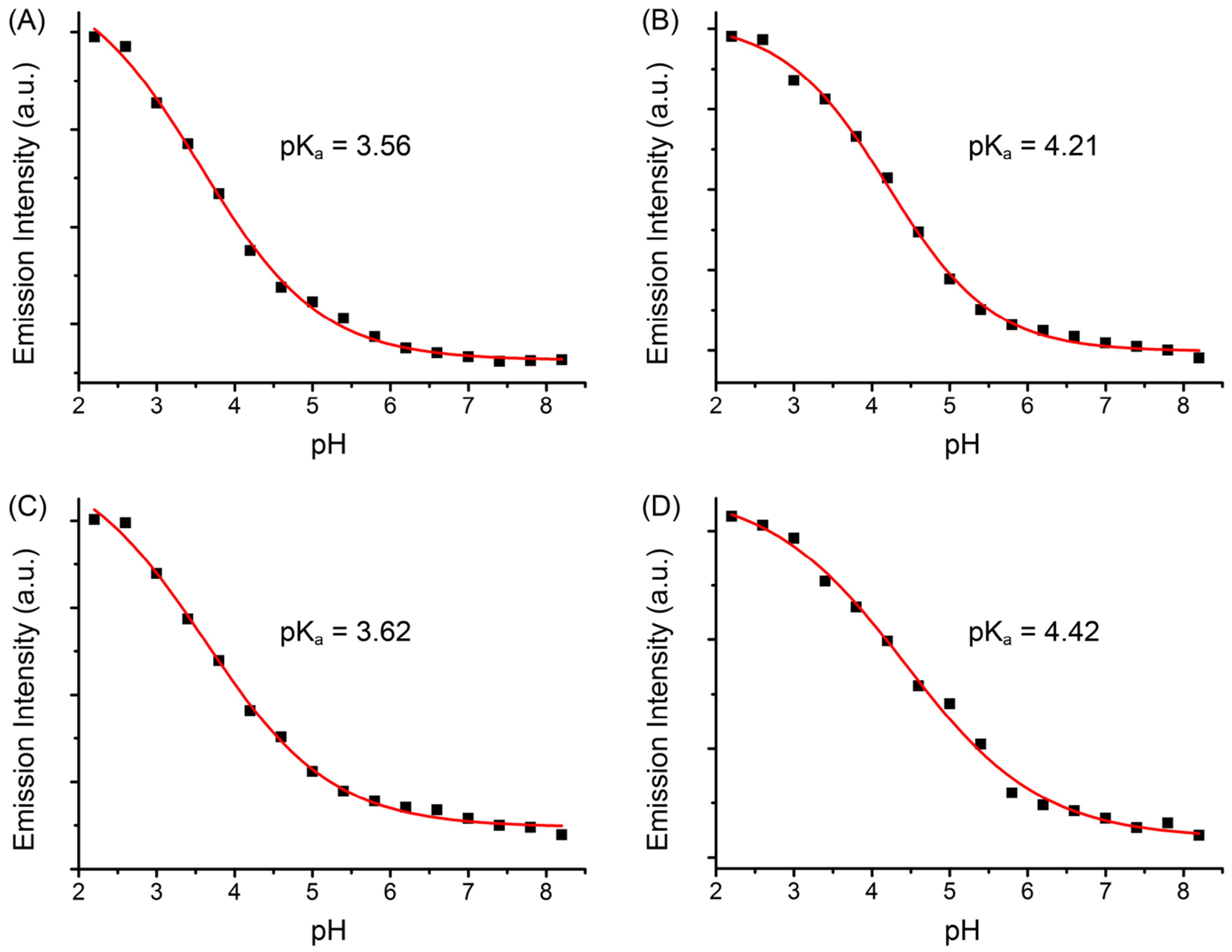

Fig. S10 Plots of emission intensity of (A) **1**, (B) **2**, (C) **3** and (D) **4** at 550, 580, 545 and 580 nm versus different pH values, respectively.

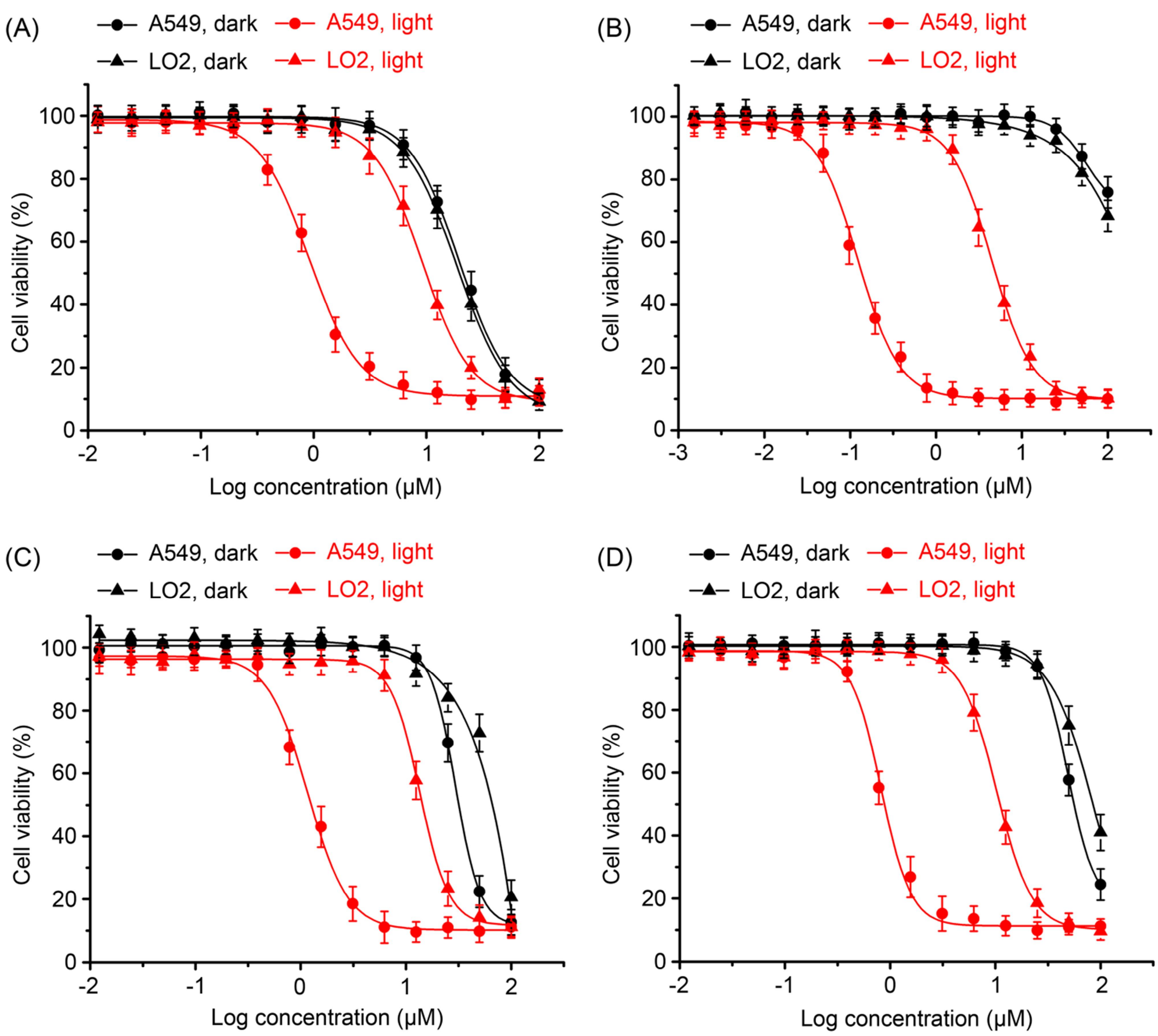

Fig. S11 In vitro PDT dose-response curves for (A) **1**, (B) **2**, (C) **3** and (D) **4** in A549 and LO2 cells. Dark and light conditions were identical except that the PDT-treated samples were irradiated with visible light (425 nm) for 15 min (36 J cm^{-2}). Data were presented as mean ± SD of three repeated experiments (n = 3).

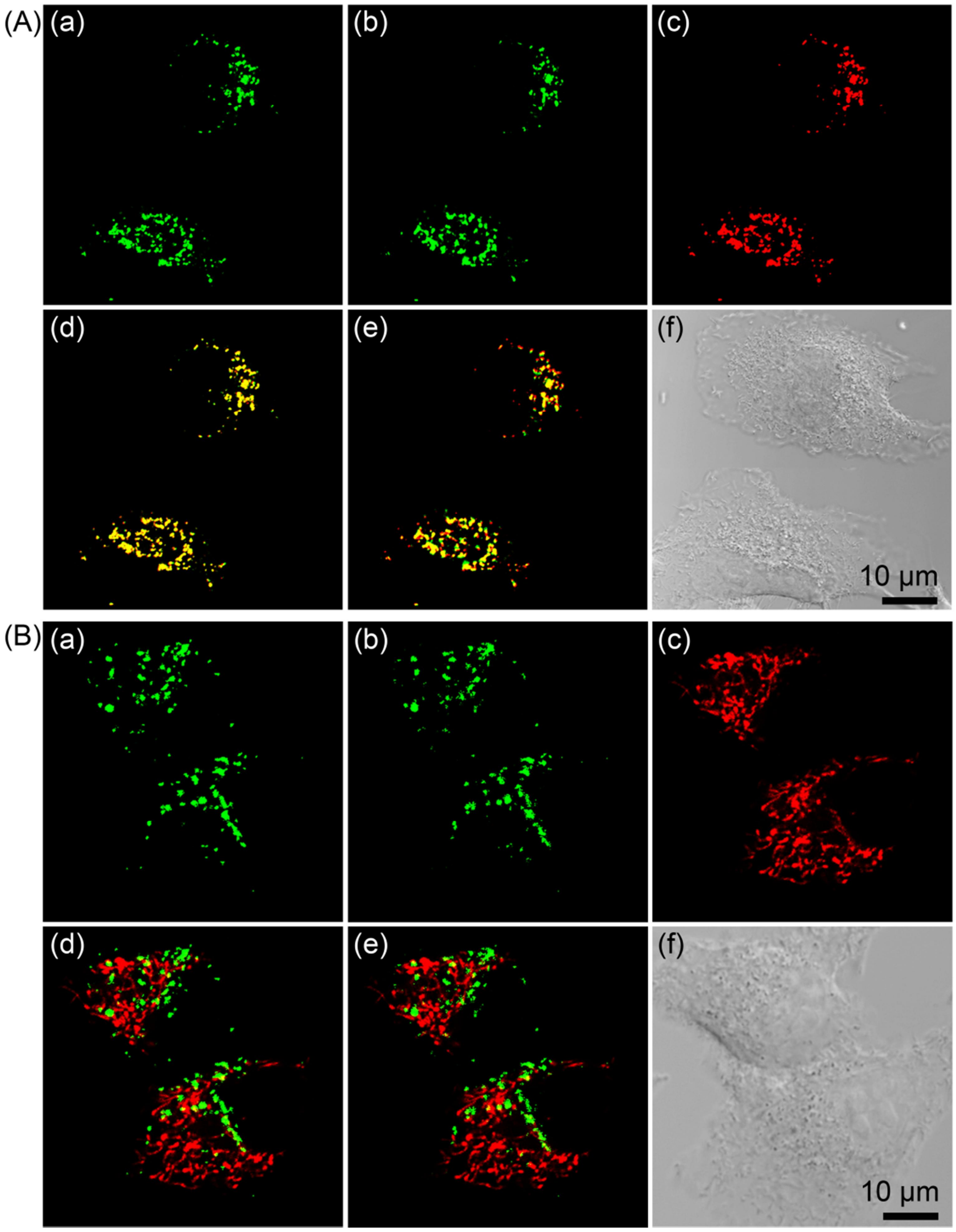

Fig. S12 (A) One- and two-photon excited phosphorescent images of A549 cells co-labeled with **1** (20 μM, 5 h) and LTDR (150 nM, 0.5 h). (a) One-photon excited **1** (λ_{ex} = 405 nm, λ_{em} = 550 ± 30 nm). (b) Two-photon excited **1** (λ_{ex} = 810 nm, λ_{em} = 550 ± 30 nm). (c) LTDR (λ_{ex} = 633 nm, λ_{em} = 668 ± 20 nm). (d) Overlay of (a) and (c). (e) Overlay of (b) and (c). (f) Bright field. (B) One- and two-photon excited phosphorescent images of A549 cells co-labeled with **1** (20 μM, 5 h) and MTDR (150 nM, 0.5 h). (a) One-photon excited **1** (λ_{ex} = 405 nm, λ_{em} = 550 ± 30 nm). (b) Two-photon excited **1** (λ_{ex} = 810 nm, λ_{em} = 550 ± 30 nm). (c) MTDR (λ_{ex} = 633 nm, λ_{em} = 665 ± 20 nm). (d) Overlay of (a) and (c). (e) Overlay of (b) and (c). (f) Bright field.

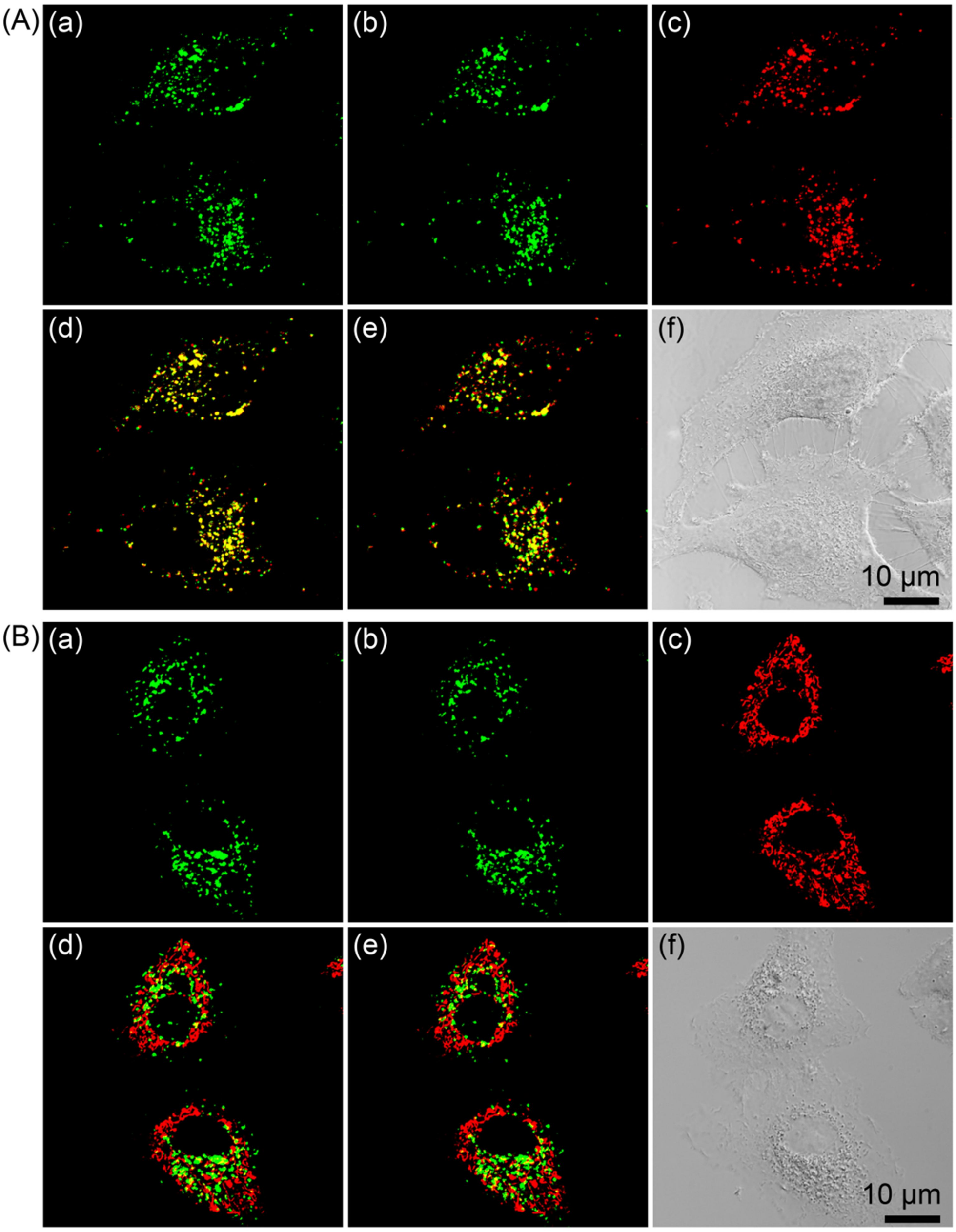

Fig. S13 (A) One- and two-photon excited phosphorescent images of A549 cells co-labeled with **3** (20 μM, 5 h) and LTDR (150 nM, 0.5 h). (a) One-photon excited **3** (λ_{ex} = 405 nm, λ_{em} = 530 ± 30 nm). (b) Two-photon excited **3** (λ_{ex} = 810 nm, λ_{em} = 530 ± 30 nm). (c) LTDR (λ_{ex} = 633 nm, λ_{em} = 668 ± 20 nm). (d) Overlay of (a) and (c). (e) Overlay of (b) and (c). (f) Bright field. (B) One- and two-photon excited phosphorescent images of A549 cells co-labeled with **3** (20 μM, 5 h) and MTDR (150 nM, 0.5 h). (a) One-photon excited **3** (λ_{ex} = 405 nm, λ_{em} = 530 ± 30 nm). (b) Two-photon excited **3** (λ_{ex} = 810 nm, λ_{em} = 530 ± 30 nm). (c) MTDR (λ_{ex} = 633 nm, λ_{em} = 665 ± 20 nm). (d) Overlay of (a) and (c). (e) Overlay of (b) and (c). (f) Bright field.

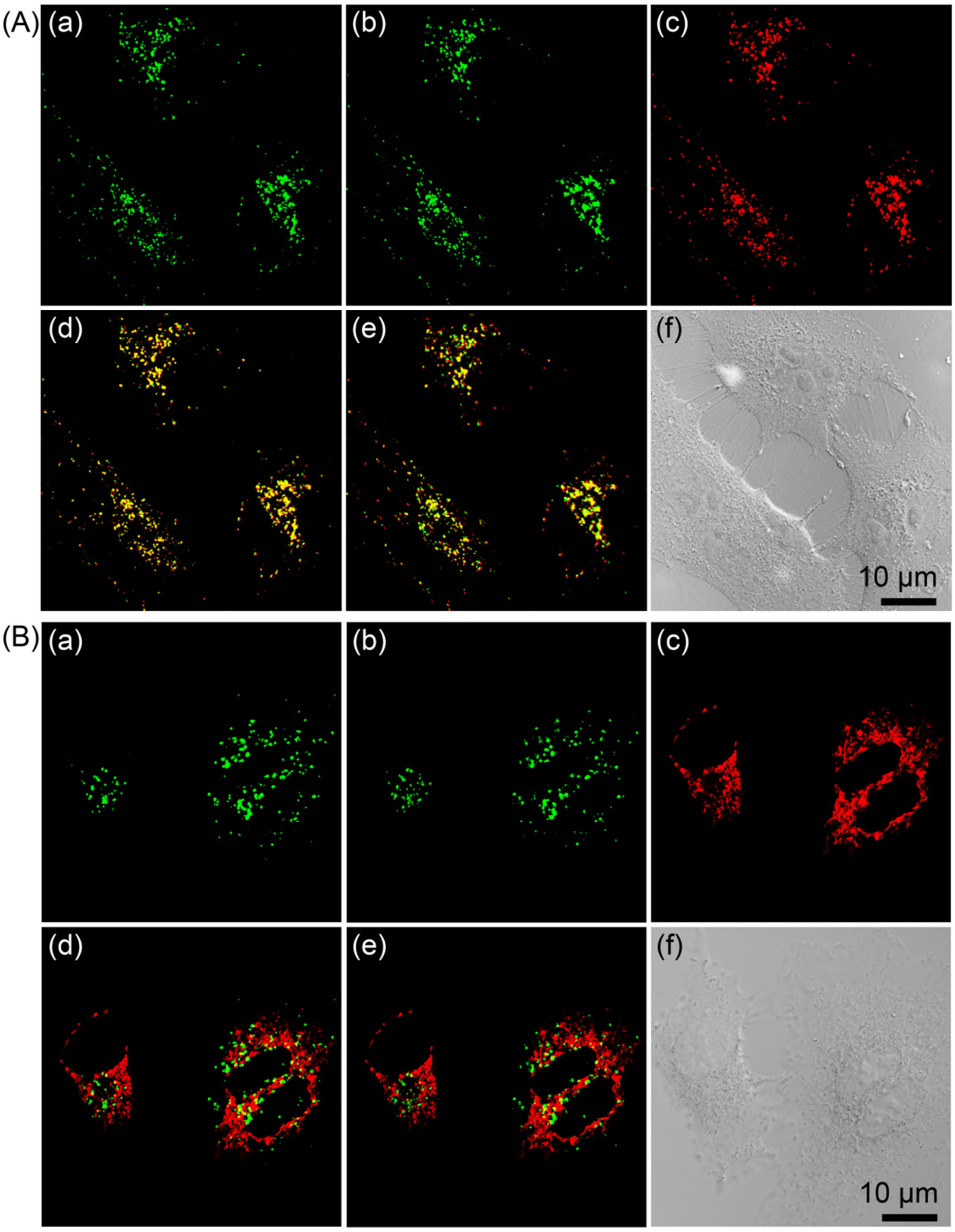

Fig. S14 (A) One- and two-photon excited phosphorescent images of A549 cells co-labeled with **4** (20 μM, 5 h) and LTDR (150 nM, 0.5 h). (a) One-photon excited **4** (λ_{ex} = 405 nm, λ_{em} = 570 ± 30 nm). (b) Two-photon excited **4** (λ_{ex} = 810 nm, λ_{em} = 570 ± 30 nm). (c) LTDR (λ_{ex} = 633 nm, λ_{em} = 668 ± 20 nm). (d) Overlay of (a) and (c). (e) Overlay of (b) and (c). (f) Bright field. (B) One- and two-photon excited phosphorescent images of A549 cells co-labeled with **4** (20 μM, 5 h) and MTDR (150 nM, 0.5 h). (a) One-photon excited **4** (λ_{ex} = 405 nm, λ_{em} = 570 ± 30 nm). (b) Two-photon excited **4** (λ_{ex} = 810 nm, λ_{em} = 570 ± 30 nm). (c) MTDR (λ_{ex} = 633 nm, λ_{em} = 665 ± 20 nm). (d) Overlay of (a) and (c). (e) Overlay of (b) and (c). (f) Bright field.

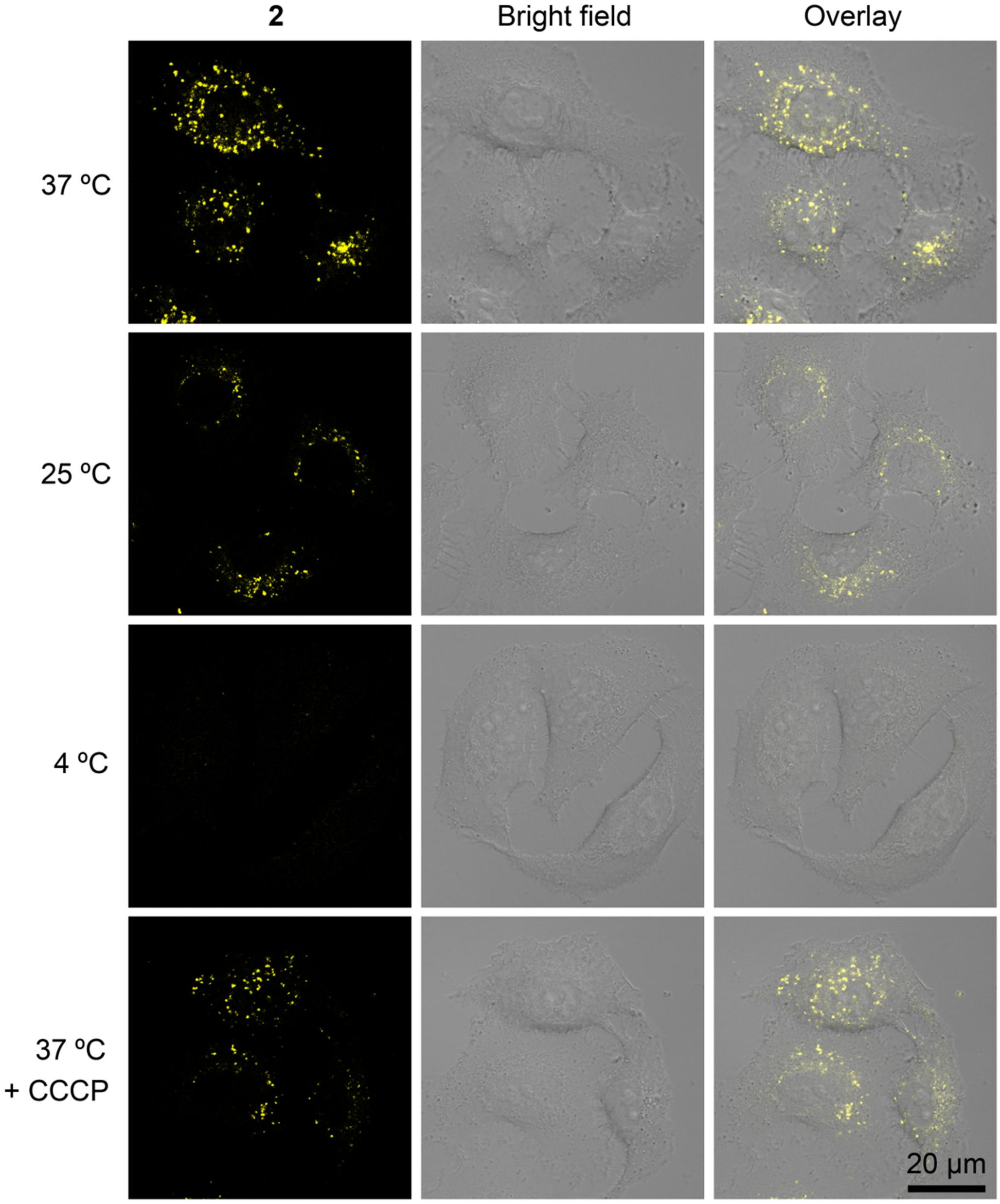

Fig. S15 Confocal images of A549 cells incubated with **2** (20 μM, λ_{ex} = 405 nm, λ_{em} = 580 ± 30 nm) for 2 h at 37 ºC (the 1st line), at 25 ºC (the 2nd line), at 4 ºC (the 3rd line), or at 37 ºC with CCCP (20 μM, 1 h) pretreatment (the 4th line).

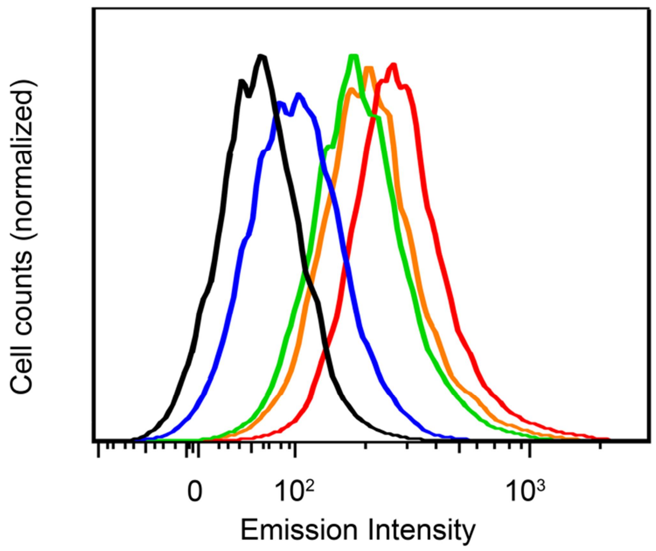

Condition	Intensity
Control	67.1
4 ºC	99.5
25 ºC	210
37 ºC	318
37 ºC, CCCP	239

Fig. S16 Flow-cytometric results of A549 cells incubated with blank medium (black), **2** (20 μM) for 2 h at 37 ºC (red), at 25 ºC (green), at 4 ºC (blue), or at 37 ºC with CCCP (20 μM, 1 h) pretreatment (orange).

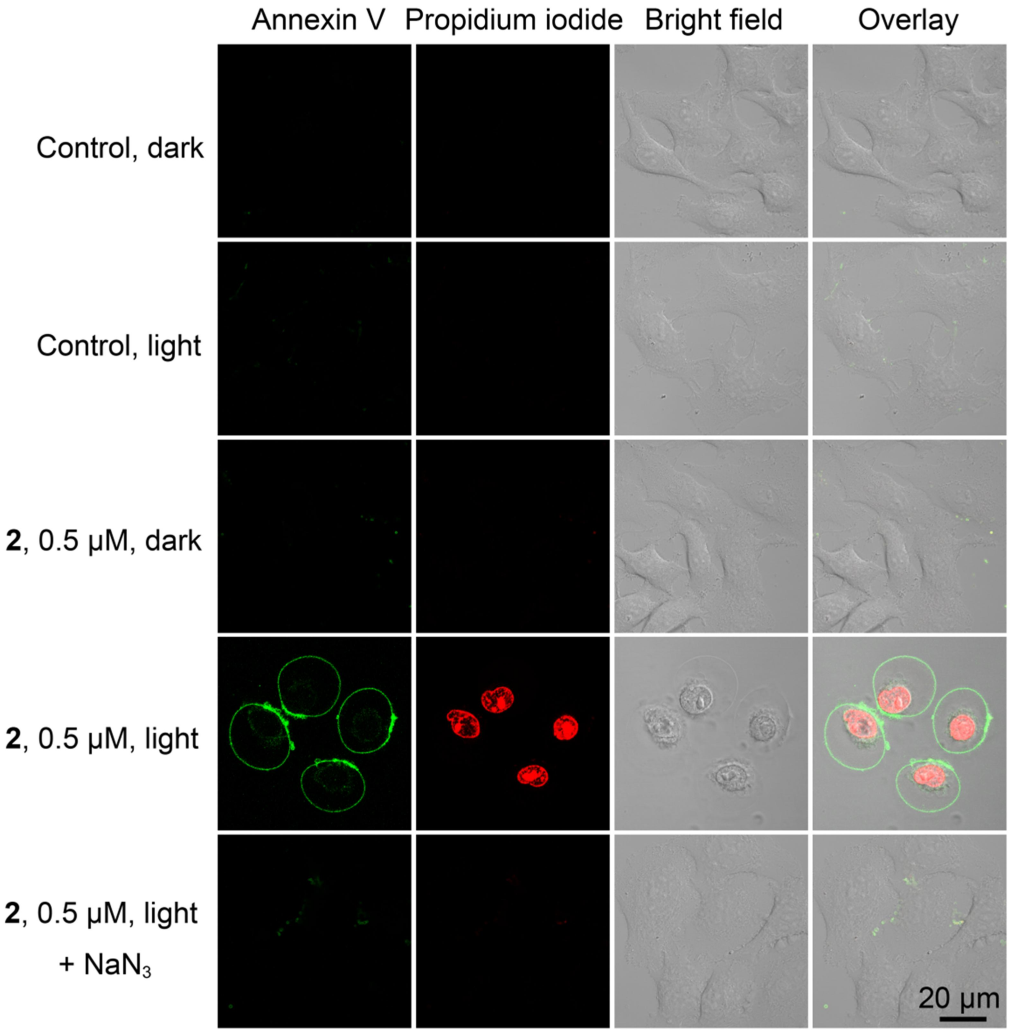

Fig. S17 Detection of apoptosis in A549 cells stained with annexin V and propidium iodide by confocal microscopy after PDT treatment with **2** (0.5 μM, 24 h) in the absence or presence of light. After A549 cells were incubated with vehicle or **2** for 12 h, the cells were irradiated with a 425 nm LED light for 15 min (36 J cm^{-2}).

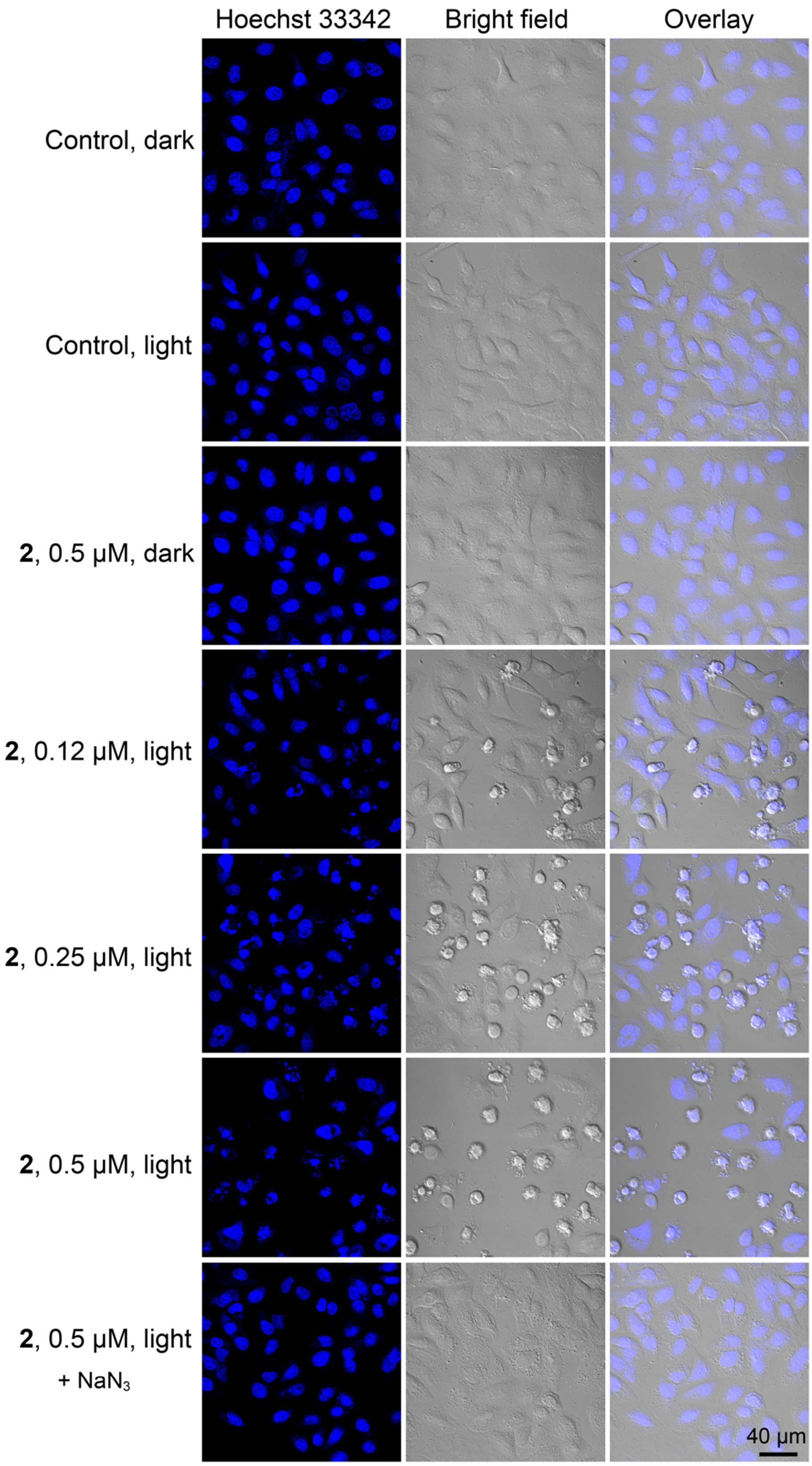

Fig. S18 Characterization of apoptosis induced by **2**-mediated PDT using Hoechst 33342 staining and confocal microscopy in the absence or presence of light. After A549 cells were incubated with vehicle or **2** for 12 h, the cells were irradiated with a 425 nm LED light for 15 min (36 J cm^{-2}).

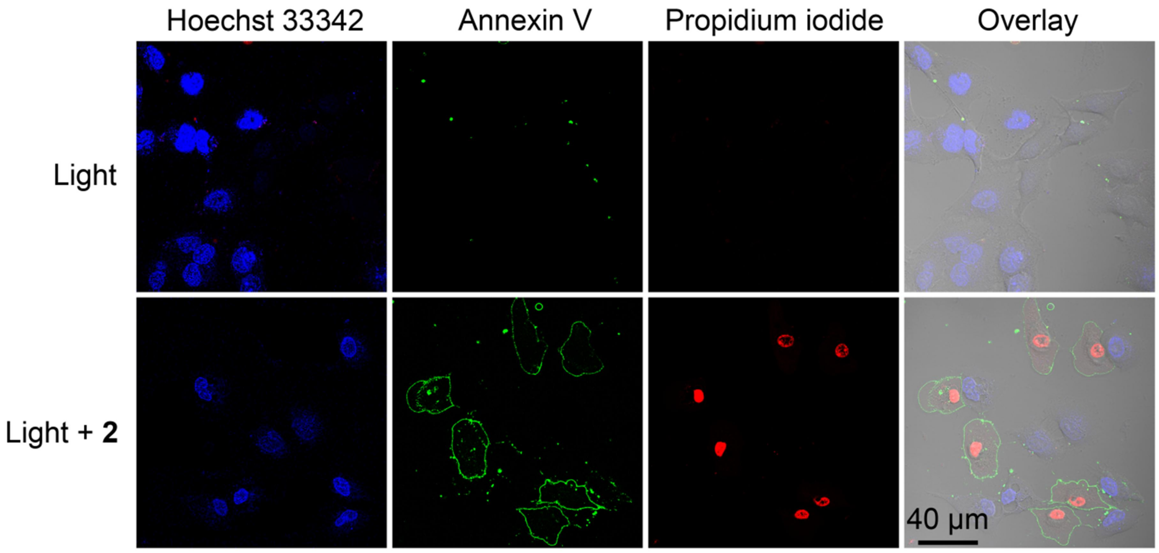

Fig. S19 Selective induction of apoptosis by **2**-mediated PDT in a A549/LO2 co-culture cell model as determined by annexin V/propidium iodide-Hoechst 33342 co-staining assay.

Table S1 Crystallographic data of (**L2**)$_2$•$(CH_3)_2CO$ and **4**•$2CH_3CH_2OH$

Compound	(**L2**)$_2$•$(CH_3)_2CO$	**4**•$2CH_3CH_2OH$
CCDC no.	883318	1061866
Empirical formula	$(C_{18}H_{12}N_4)_2$•C_3H_6O	$C_{40}H_{24}N_6F_{10}PIr$•$2C_2H_6O$
Molecular weight	626.71	1093.96
Description	Block, colorless	Block, yellow
Temperature (K)	173 K	150 K
λ (Å)	0.71073	0.71073
Crystal system	monoclinic	triclinic
Space group	P21/c	P-1
a (Å)	11.4678(17)	8.5758(12)
b (Å)	19.447(3)	14.436(2)
c (Å)	15.154(2)	17.199(3)
α (^{o})	90	79.430(4)
β (^{o})	112.202(2)	89.672(4)
γ (^{o})	90	84.686(5)
Volume, $Å^3$	3129.0(8)	2084.0(5)
Z	4	2
μ/mm^{-1}	0.084	3.334
F(000)	1312.0	1080.0
θ_{max} (deg)	27.034	27.472
Completeness to θ_{max}	0.987	0.984
Density (calcd) (mg/m^{-3})	1.330	1.743
[R_{int}]	0.0232	0.0982
Reflections collected/unique	18271/6784	19436/9390
$R1^a$[I > 2σ(I)]	0.0395	0.0585
$wR2^a$	0.1068	0.1128
GOF^b	1.039	0.992

$^aR1 = \sum\|F_0|-|F_c\|/\sum|F_0|, wR2 = \left\{\sum\left[w\left(F_0^2-F_c^2\right)^2\right]/\sum\left[w\left(F_0^2\right)^2\right]\right\}^{1/2}$ $^bGOF = \left\{\sum\left[w\left(F_0^2-F_c^2\right)^2/(n-p)\right]\right\}^{1/2}$

where *n* is the number of data and *p* is the number of parameters refined.

Table S2 Selected bond lengths (Å) and bond angles (deg) of (**L2**)$_2$•$(CH_3)_2CO$ and **4**•$2CH_3CH_2OH$

Compoud	(**L2**)$_2$•$(CH_3)_2CO$		**4**•$2CH_3CH_2OH$	
bond lengths (Å)	C1–N1	1.3887(17)	Ir1–N1	2.049(5)
	C7–N1	1.3221(17)	Ir1–N2	2.043(5)
	C6–N2	1.3774(18)	Ir1–N3	2.114(5)
	C7–N2	1.3622(16)	Ir1–N5	2.161(4)
	C8–N3	1.3398(17)	Ir1–C1	1.996(5)
	C12–N3	1.3484(18)	Ir1–C12	1.990(6)
	C9–N4	1.3705(16)		
	C18–N4	1.3829(16)		
bond angles (deg)	C1–N1–C7	104.40(11)	C1–Ir1–N1	80.2(2)
	C6–N2–C7	106.48(11)	C12–Ir1–N1	96.5(2)
	C8–N3–C12	118.87(12)	N2–Ir1–N1	173.19(16)
	C9–N4–C18	108.63(11)	C1–Ir1–N3	97.9(2)
	N1–C7–N2	113.52(12)	N2–Ir1–N3	97.68(18)
	C7–C8–N3	117.83(11)	N5–Ir1–N3	76.11(18)

Table S3 (Photo)cytotoxicity (IC_{50}, μM) of the tested compounds toward MCF-7 cells

Compound	MCF-7		
	Dark[a]	Light[b]	PI[c]
**1**	19.1 ± 3.0	0.87 ± 0.06	22.0
**2**	>100	0.13 ± 0.02	>769
**3**	46.9 ± 5.7	0.95 ± 0.10	49.4
**4**	59.8 ± 6.4	0.36 ± 0.05	166
**L1**	83.3 ± 7.3	5.1 ± 0.6	16.3
**L2**	>100	>100	-
**P1**	23.9 ± 3.1	5.6 ± 0.4	4.3
**P2**	12.5 ± 2.0	2.4 ± 0.3	5.2
Cisplatin	25.1 ± 2.0	23.5 ± 2.2	1.1

[a] Cells were incubated with the indicated compounds for 48 h. [b] Cells were incubated with the indicated compounds for 12 h and then irradiated with a 425 nm LED light array for 15 min (36 J cm^{-2}). [c] PI (phototoxicity index) is the ratio of the IC_{50} value in the dark to that obtained upon light irradiation.

Table S4 Lipophilicity and cellular uptake efficiency of complexes **1−4**

Complex	Lipophilicity (log $P_{o/w}$)[a]	Amount of iridium[b] (ng/10^6 cells)	
		5 h	12 h
**1**	1.97	320.5 ± 25.2	479.1 ± 32.9
**2**	2.07	151.3 ± 19.3	240.3 ± 17.6
**3**	2.01	244.7 ± 30.5	306.7 ± 15.1
**4**	2.12	143.3 ± 21.2	210.6 ± 22.7

[a] Log $P_{o/w}$ is defined as the logarithmic ratio of Ir(III) concentration in *n*-octanol to that in the aqueous phase. [b] Amount of iridium in A549 cells was determined by ICP-MS after cells were incubated with iridium(III) complexes (20 μM) at 37 °C for 5 or 12 h.

Supporting references

1. L. He, S. Y. Liao, C. P. Tan, R. R. Ye, Y. W. Xu, M. Zhao, L. N. Ji and Z. W. Mao, *Chem. –Eur. J.*, 2013, **19**, 12152–12160.
2. C. Y. Li, M. X. Yu, Y. Sun, Y. Q. Wu, C. H. Huang and F. Y. Li, *J. Am. Chem. Soc.*, 2011, **133**, 11231–11239.
3. G. M. Sheldrick, *Acta Crystallogr. A*, 2008, **64**, 112–122.
4. L. He, C. P. Tan, R. R. Ye, Y. Z. Zhao, Y. H. Liu, Q. Zhao, L. N. Ji and Z. W. Mao, *Angew. Chem., Int. Ed.*, 2014, **53**, 12137–12141.
5. N. S. Makarov, M. Drobizhev and A. Rebane, *Opt. Express*, 2008, **16**, 4029-4047.
6. C. Xu and W. W. Webb, *J. Opt. Soc. Am. B*, 1996, **13**, 481–491.
7. B. A. Lindig, M. A. J. Rodgers and A. P. Schaap, *J. Am. Chem. Soc.*, 1980, **102**, 5590–5593
8. J. M. Wessels, C. S. Foote, W. E. Ford and M. A. Rodgers, *Photochem. Photobiol.*, 1997, **65**, 96–102.
9. M. J. McKeage, S. J. Berners-Price, P. Galettis, R. J. Bowen, W. Brouwer, L. Ding, L. Zhuang and B. C. Baguley, *Cancer Chemother. Pharmacol.*, 2000, **46**, 343–350.

Movies

Movie S1. Real-time monitoring of lysosomal integrity in A549 cells loaded with **2** (10 μM, 12 h) after PDT treatment (120 s, 4.8 J cm^{-2}). Cell imaging was then carried out immediately by confocal microscopy and photographs were taken every 5 min. Emission was collected at 580 ± 30 nm upon excitation at 405 nm.

Movie S2. Real-time monitoring of lysosomal integrity in A549 cells loaded with **2** (10 μM, 12 h) without PDT treatment. Cell imaging was then carried out immediately by confocal microscopy and photographs were taken every 5 min. Emission was collected at 580 ± 30 nm upon excitation at 405 nm.
